# Iron(III) Complexes with Non-Steroidal Anti-Inflammatory Drugs: Structure, Antioxidant and Anticholinergic Activity, and Interaction with Biomolecules

**DOI:** 10.3390/ijms24076391

**Published:** 2023-03-28

**Authors:** Filitsa Dimiza, Amalia Barmpa, Antonios Chronakis, Antonios G. Hatzidimitriou, Yiannis Sanakis, Athanasios N. Papadopoulos, George Psomas

**Affiliations:** 1 Department of General and Inorganic Chemistry, Faculty of Chemistry, Aristotle University of Thessaloniki, GR-54124 Thessaloniki, Greece; fildimchem@yahoo.gr (F.D.); emili.b19@gmail.com (A.B.); hatzidim@chem.auth.gr (A.G.H.); 2 Department of Nutritional Sciences and Dietetics, International Hellenic University, GR-57400 Thessaloniki, Greece; antonischronakis@yahoo.com (A.C.); papadnas@ihu.gr (A.N.P.); 3 Institute of Nanoscience and Nanotechnology, National Centre of Scientific Research “Demokritos”, GR-15310 Agia Paraskevi, Greece; i.sanakis@inn.demokritos.gr

**Keywords:** iron(III) complexes, non-steroidal anti-inflammatory drugs, antioxidant activity, anticholinergic activity, interaction with albumins, interaction with DNA

## Abstract

One the main research goals of bioinorganic chemists is the synthesis of novel coordination compounds possessing biological potency. Within this context, three novel iron(III) complexes with the non-steroidal anti-inflammatory drugs diflunisal and diclofenac in the presence or absence of the nitrogen donors 1,10-phenanthroline or pyridine were isolated and characterized by diverse techniques. The complexes were evaluated for their ability to scavenge in vitro free radicals such as hydroxyl, 1,1-diphenyl-2-picrylhydrazyl and 2,2′-azino-bis(3-ethylbenzothiazoline-6-sulfonic acid) radicals, revealing their selective potency towards hydroxyl radicals. The in vitro inhibitory activity of the complexes towards the enzymes acetylcholinesterase and butyrylcholinesterase was evaluated, and their potential to achieve neuroprotection appeared promising. The interaction of the complexes with calf-thymus DNA was examined in vitro, revealing their ability to intercalate in-between DNA nucleobases. The affinity of the complexes for serum albumins was evaluated in vitro and revealed their tight and reversible binding.

## 1. Introduction

The biological importance of iron has been known since ancient times. Iron is the most abundant transition metal ion in the human body and is found in small amounts [1]. It is mainly found in the active center of proteins and enzymes, such as the hemoproteins hemoglobin, myoglobin and cytochromes, or the iron–sulfur proteins and ferritin [2]. Iron is found in two oxidation states (+2 and +3), and its ability to interconvert between these two states makes it crucial for important biochemical reactions but also dangerous, due to its involvement in undesired reactions [3]. The main biological functions of iron are the transportation of oxygen and electrons, influencing cellular metabolism, respiration and DNA synthesis [4,5,6], photosynthesis [7], and other basic cellular processes in the body, all contributing to good health and proper functioning [3,5]. Despite its beneficial effect, an excess of iron may generate free radicals, resulting in tissue damage [8], and its accumulation may be a reason for tumors and other cancers [9,10,11]. In addition, a malfunction of iron homeostasis may result in iron deficiency or iron overload, which may be related to heart failure [12], brain aging, and neurodegenerative diseases such as Alzheimer’s disease (AD), Parkinson’s disease, multiple sclerosis [13,14], and respiratory diseases [15]. As a biological elemental, iron has drawn the attention of bioinorganic chemists regarding the potential activity of its compounds. Iron oxide nanoparticles have been reported for their antimicrobial and cytotoxic activity [6,16], while iron complexes have shown anticancer [17], antimicrobial [18,19,20], and antioxidant [18,21,22] activities.

Alzheimer’s disease is one of the most prevalent neurodegenerative diseases and the major cause of dementia in the elderly. It causes memory loss, behavioral changes, and progressive decline in mental and functional abilities [23]. According to more recent estimates, approximately 50 million people were living with AD worldwide in 2020, and this number is projected to reach 152 million by 2050 [24]. To explain this multifactorial disease, several hypotheses have been proposed and pursued. The design of cholinesterase inhibitors based on the central cholinergic hypothesis is the most popular clinical strategy for the treatment of AD so far [25]. In the central nervous system, acetylcholine (ACh) has multiple roles. ACh is the main neurotransmitter of the nervous system and the one that maintains communication between neurons. It is known to play an important role in memory and learning and is in abnormally short supply in ailing brains [26,27]. ACh deficits lead to neuronal and synaptic dysfunction, resulting in dementia [26,27]. Acetylcholine is rapidly destroyed by the enzyme acetylcholinesterase (AChE) and thus is effective only briefly. Inhibitors of the enzyme (drugs known as anticholinesterases) prolong the lifetime of ACh. Such agents include physostigmine, neostigmine, and tacrine, which are used in the treatment of Alzheimer’s disease, amongst other diseases [28].

AD is a complex neurological disorder that is characterized by progressive cognitive decline and the loss of brain cells. While the exact causes of AD are not fully understood, one theory that has gained significant attention in recent years is the role of oxidative stress, which includes elevated levels of reactive oxygen species (ROS) [29]. ROS are molecules that can cause damage to cells, including brain cells, and are thought to contribute to the process of neurodegeneration by promoting inflammation and damaging cellular structures in the brain. Several studies have shown that levels of ROS are elevated in the brains of people with AD, and that these levels correlate with the severity of cognitive impairment [30,31]. Additionally, animal studies have suggested that treatment with antioxidants, which can neutralize ROS, may help to prevent or slow the progression of AD [32]. Overall, as oxidative stress appears to play an important role in the etiology of AD, targeting ROS with antioxidants or other free radical scavengers may have a critical role in the treatment of AD, leading to effective drugs being developed. While the evidence for this theory is still emerging, it offers a promising avenue for further research and the development of novel therapies for AD. Moreover, a number of inflammatory markers have been identified in AD brain tissue [33]. Research indicates that inflammation plays a significant role in the development of senile plaques (SPs), a marker of AD [34]. Non-steroidal anti-inflammatory drugs (NSAIDs), for example, are expected to slow the progression of the disease by interfering with SP formation or suppressing the inflammation associated with SPs [35]. In fact, various epidemiologic studies have been conducted to support the notion that NSAIDs may be beneficial for the management of Alzheimer’s disease.

**Figure 1:**
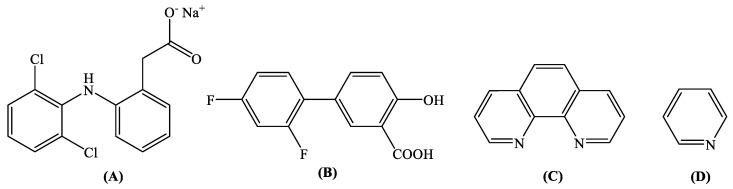
Syntax formula of (**A**) sodium diclofenac (Nadicl), (**B**) diflunisal (H_2_difl), (**C**) 1,10-phenanthroline (phen), and (**D**) pyridine (py).

Sodium diclofenac (Nadicl) (Figure 1A) is an analgesic, antipyretic, and anti-inflammatory agent [36] proposed for the treatment of rheumatoid arthritis and osteoarthritis [37,38]. However, recent studies have shown that the use of sodium diclofenac may increase the cardiovascular problems when compared to paracetamol and other traditional NSAIDs [39]. As a typical NSAID, sodium diclofenac has been proven to be a better medication than paracetamol for the treatment of COVID-19, regarding its analgesic and antipyretic efficacy and the enhancement of the immune response of patients [40,41]. Considering the coordination compounds containing diclofenac ligands, a series of copper(II) [38,42,43,44,45], cobalt(II) [45], manganese(II/III) [46,47,48], cadmium(II) [49], tin(IV) [50], nickel(II) [48,51], Zn(II) [52,53], and Ag(I) [54] have been found in the literature, with their biological profile investigated in most cases [38,42,43,44,45,46,48,51,52].

Diflunisal (H_2_difl, Figure 1B) is a potent difluoro analog of aspirin and, as a typical NSAID, shows analgesic and anti-inflammatory activity [55]. Because of its relatively long half-life period of activity (~12 h), diflunisal is often used to alleviate acute pain resulting from oral surgery, such with as the removal of wisdom teeth [56]. Diflunisal is also recommended for the chronic treatment of symptoms of arthritis [57]. Recent studies have reported that diflunisal can be safely administered to selected patients suffering from transthyretin amyloidosis cardiomyopathy, since it regulates some parameters of cardiac structure and function [58,59], and, because of its low price, may be used as an alternative to the more expensive drug tafamidis [60]. Furthermore, a series of first-row transition metal complexes of diflunisal (i.e., Cu(II) [61], Co(II) [62], Ni(II) [63], and Zn(II) [64]) have also been reported for their antioxidant potency and their interaction with biomacromolecules, as well as two tin(IV) complexes showing antimicrobial activity [65,66], a series of Bi(III) complexes active towards *Leishmania major* [67,68], and a Ga(III) complex studied for its in vitro cytotoxic activity [69].

In the context of the importance and extended use of NSAIDs in medicine and the enhanced activity reported for their metal complexes, as well as the biological relevance of iron and as a continuation of our research concerning transition metal complexes of the NSAIDs diflunisal and diclofenac [42,43,46,51,52,61,62,63,64,70,71,72,73,74], we have synthesized and characterized three novel Fe(III) complexes with the NSAIDs diflunisal and diclofenac in the presence or absence of the nitrogen donors 1,10-phenanthroline (phen) and pyridine (py) as co-ligands (Figure 1C,D). The resultant complexes [Fe_2_(difl)_2_(MeO)_2_(phen)_2_]∙H_2_O (complex **1**), [Fe_3_O(dicl)_6_(py)_3_]Cl∙py (complex **2**), and [Fe_3_O(dicl)_6_(MeOH)_3_][FeCl_4_]·Hdicl·1.5MeOH∙H_2_O (complex **3**) were characterized by physicochemical and spectroscopic (infrared, electronic, Mössbauer, and EPR) techniques, and their structures were determined by single-crystal X-ray crystallography.

In addition to the synthesis and the structural characterization of complexes **1**–**3**, the main goal of the current research is focused on the assessment of the potential biological activity of the complexes, including their antioxidant capacity and their inhibitory effectiveness towards cholinesterase enzymes. The application of NSAIDs and their compounds as analgesic, anti-inflammatory, and anticholinergic medications is often related to free radicals scavenging [22,74,75,76,77]. Therefore, the antioxidant capacity of the complexes was evaluated in vitro by determining their ability to scavenge 1,1-diphenyl-picrylhydrazyl (DPPH), 2,2′-azino-bis(3-ethylbenzothiazoline-6-sulfonic acid) (ABTS), and hydroxyl radicals. Bearing in mind that AChE and butyrylcholinesterase (BuChE) play a key role in the regulation of acetylcholine levels in the brain, and their inhibition can lead to increased acetylcholine levels that can have potential therapeutic benefits in certain neurodegenerative diseases, such as AD, the anticholinergic ability of the compounds was studied by evaluating their activity to inhibit in vitro the two metabolic enzymes of acetylcholine, AChE and BuChE, as a means to check whether they may serve as potential neuroprotectors.

Further biological studies of complexes **1**–**3** were focused on their interaction with calf-thymus (CT) DNA and their affinity for bovine serum albumin (BSA) and human serum albumin (HSA). DNA is often a potential biological target [78]. The interaction of the complexes with CT DNA was investigated by UV-vis spectroscopy and viscosity measurements and via competitive studies with ethidium bromide (EB) using fluorescence emission spectroscopy. Albumins are blood serum proteins involved in the transportation of drugs and small molecules through the blood stream [79,80,81]. The affinity of the compounds for BSA and HSA was monitored by fluorescence emission spectroscopy. A strong binding of the complexes to these biomacromolecules may offer enhanced or differentiated biological properties.

## 2. Results and Discussion

### 2.1. Synthesis of the Complexes

The synthesis of complex **1** in a high yield was achieved via the aerobic reaction of the dianion of diflunisal (difl^−2^), which was formed via the deprotonation of H_2_difl by KOH in a 1:2 H_2_difl:KOH ratio, with a solution of FeCl_3_∙6H_2_O (in a 1:1 Fe^3+^:difl^−2^ ratio) in the presence of the N,N’-donor phen as a co-ligand. Complexes **2**–**3** were prepared via the aerobic reaction of FeCl_3_∙6H_2_O with sodium diclofenac in methanol in a 1:2 Fe^3+^:dicl^−1^ ratio in the absence (for **2**) or presence of pyridine (for **3**). The characterization of the three resultant complexes was performed by IR, UV-vis, Mössbauer, and EPR spectroscopies, as well as single-crystal X-ray crystallography.

The complexes are air-stable, soluble mainly in DMSO and DMF and insoluble in most organic solvents and H_2_O. The molar conductivity value for complex **1** (Λ_M_ = 5 S·cm^2^·mol^−1^ for 1-mM DMSO solution) may indicate its non-electrolytic nature. On the other hand, in the case of complexes **2** and **3**, the Λ_M_ values (65–70 S·cm^2^·mol^−1^ in a 1-mM DMSO solution) are indicative of 1:1 electrolytes and may subsequently suggest their integrity in the solution [82].

### 2.2. Structure of the Complexes

The crystal structures of complexes **1**–**3** were determined by single-crystal X-ray crystallography. Complex **1** is a neutral dinuclear complex, while complexes **2** and **3** contain a cationic trinuclear basic carboxylate complex (show structural similarities and differences) which are neutralized by a chlorido anion and a [tetrachloroiron(III)] anionic complex, respectively. Crystallographic data for complexes **1**–**3** are presented in Table S1.

#### 2.2.1. Crystal Structure of Complex **1**

**Figure 2:**
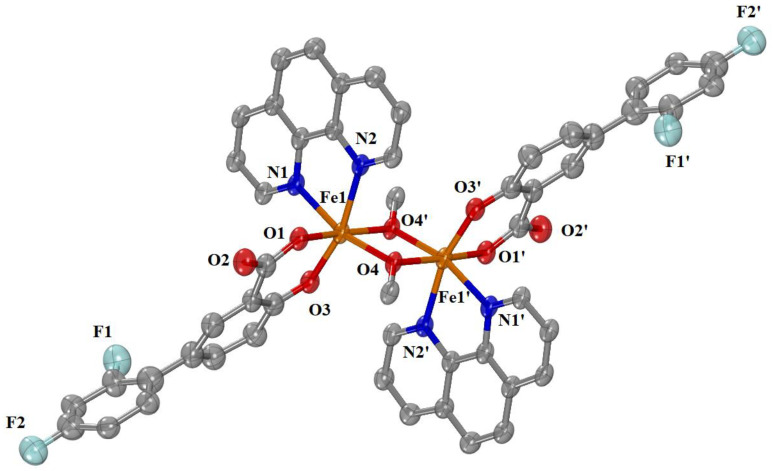
Molecular structure of complex **1**. Aromatic and methyl hydrogen atoms and water solvate molecules are omitted for clarity. Symmetry: (′) −x + 1, −y + 1, −z + 1. (Atom color code: C in grey; O in red; N in blue; Fe in orange; F in light blue-green).

Complex **1** crystallizes in the triclinic system and P–1 space group. The molecular structure of the complex is shown in Figure 2. Selected bond distances and angles are cited in Table 1 and Table S2. A water solvate molecule is also present.

It is a neutral dinuclear Fe(III) complex where the Fe(III) ions are bridged by the oxygen atoms of two methoxy groups. The structure is centrosymmetric (the center of the symmetry is located in the middle of the distance between the two Fe(III) ions), so the description is discussed in terms of one iron(III) ion. Each Fe(III) ion is six-coordinated with a FeN_2_O_4_ coordination sphere and a distorted octahedral geometry. Two of the oxygen atoms come from the methoxy bridges, while the other two oxygen atoms come from the doubly deprotonated diflunisal (difl^2−^). The diflunisal ligands are bidentately bound to the Fe(III) ions via a carboxylato oxygen and the phenol oxygen atoms forming a six-membered chelate ring. The coordination sphere of each Fe(III) ion is completed by two nitrogen atoms from the phen ligand. Among the Fe–O bond lengths in the coordination sphere, the Fe–O_diflunisal_ bond lengths are the shortest in the coordination sphere (1.920 (2)–1.928 (2) Å) compared to Fe–O_methoxo_ (1.9726 (19)−1.9984 (19) Å), while the Fe–N_phen_ are the longest (2.191 (3)–2.120 (2) Å). The separation distance between the two Fe(III) ions is 3.119 Å and is in the range (3.058–3.24 Å) expected for dinuclear iron(III) complexes bearing two methoxo bridges [83,84,85,86,87,88].

To the best of our knowledge, this is the second example where diflunisal is doubly deprotonated after being coordinated in the bidentate chelating mode, as also found in a mononuclear Ga(III) complex [69]. Diflunisal is a typical salicylate derivative and may behave similarly to salicylato ligands. In addition to the typical salicylato monoanion, the double deprotonation of salicylic acid may result in either the bidentate chelating coordination of salicylato ligands leading to the formation of mononuclear complexes [89,90,91,92] or the tridentate bridging mode leading to polynuclear complexes [93,94,95,96,97].

#### 2.2.2. Crystal Structure of Complex **2**

**Figure 3:**
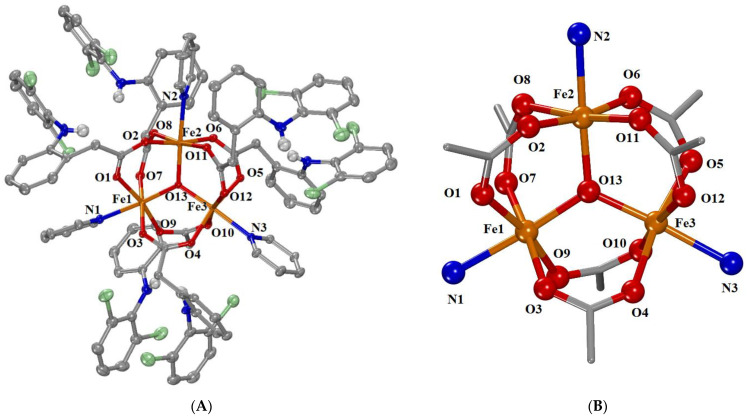
(**A**) Molecular structure of the cationic complex [Fe_3_O(μ_2_-dicl-O,O′)_6_(py)_3_]^+^. Aromatic hydrogen atoms, counter anions, and solvate molecules are omitted for clarity. (**B**) The core structure of complex [Fe_3_O(μ_2_-dicl-O,O′)_6_(py)_3_]^+^. (Atom color code: C in grey; H in white; O in red’ N in blue; Fe in orange; Cl in light green).

Complex **2** crystallizes in the triclinic system and P–1 space group. This compound is a typical cationic trinuclear Fe(III) complex with a triangular Fe_3_ arrangement, and it belongs to the class of “basic carboxylates” [22]. The molecular structure of the complex is shown in Figure 3, and selected bond distances and angles are cited in Table 2 and Table S3. One badly disordered over three positions, the solvate pyridine molecule, as well as one disordered over four positions, the chlorido anion, exist for each complex monocation.

It is a trinuclear oxo-centered iron(III) cationic complex with the formula [Fe^III^_3_(µ_3_-O)(µ-dicl-O,O′)_6_(py)_3_]^+^ and its charge is neutralized by a chlorido anion. Each iron(III) ion is six-coordinated with a FeNO_5_ coordination sphere with a distorted octahedral geometry. The six diclofenac ligands are bidentately coordinated to the Fe(III) ions, thus forming six μ_1,3_-bridges, two bridges for each pair of Fe(III) ions. The coordination sphere of each Fe is completed by the central oxo-bridging oxygen and a nitrogen atom from the pyridine ligands.

The three iron ions are arranged in an (almost) isosceles triangle with Fe…Fe interatomic distances ranging from 3.296–3.311 Å (Table 2). The three Fe(III) ions are triply bridged by an oxo atom (O13) located in the center of this triangle with the Fe–O distances being in the range of 1.890 (2)–1.922 (2) Å (Table 2), being the shortest bond distance in the coordination sphere (Fe–O_carboxylato_ = 1.995 (3)–2.041 (3) Å, Fe–N = 2.149 (3)–2.179 (3) Å). The three Fe–O–Fe angles around the central O atom (O13) range between 119.54 (12)°–120.29 (13)°, and their sum of 360° may indicate the co-planarity of the four atoms that form the core [Fe_3_(µ_3_–O)] (Figure 3).

The intraligand H-bonds developed between imino H and carboxylato O atoms of the diclofenac ligands contribute to additional stability to the structure (Table S4).

#### 2.2.3. Crystal Structure of Complex **3**

**Figure 4:**
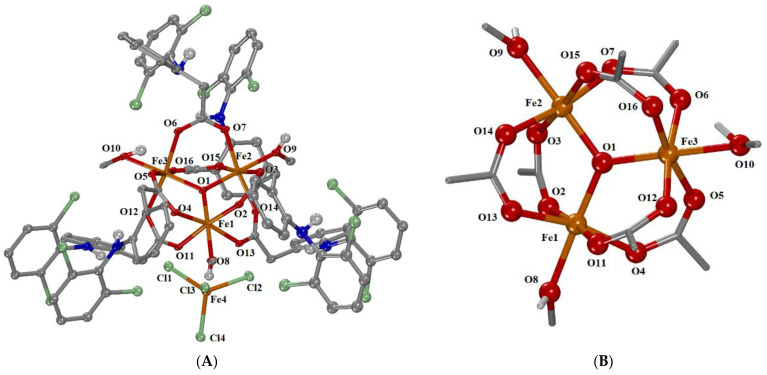
(**A**) The molecular structure of complex [Fe_3_O(μ_2_-dicl-O,O′)_6_(ΜeOH)_3_][FeCl_4_]. Aromatic and methyl hydrogen atoms as well as diclofenac acid and methanol and water solvate molecules are omitted for clarity. (**B**) The core structure of the cationic complex [Fe_3_O(μ_2_-dicl-O,O′)_6_(MeOH)_3_]^+^. (Atom color code: C in grey; H in white; O in red; N in blue; Fe in orange; Cl in light green).

Complex **3** crystallizes in the triclinic system and P–1 space group. This compound consists of the cationic trinuclear complex [Fe_3_O(μ_2_-dicl-O,O′)_6_(MeOH)_3_]^+^ and the anionic complex [FeCl_4_]^−^. A solvate diclofenac acid is also present. Furthermore, three partially disordered solvate methanol molecules, as well as two partially disordered water solvate molecules, exist in the unit cell. The molecular structure of the complex is shown in Figure 4. Selected bond distances and angles are cited in Table 3 and Table S5.

The cationic complex **3** is a trinuclear oxo-centered transition metal carboxyl complex with the formula [Fe^III^_3_(µ_3_-O)(µ-dicl-O,O′)_6_(MeOH)_3_]^+^, with a triangular Fe_3_ arrangement, and belongs to the “basic carboxylates”, like complex **2**. In the cationic complex, each six-coordinated iron(III) ion has a FeO_6_ coordination environment with a distorted octahedral geometry. Each of the six diclofenac ligands is bidentately coordinated to two Fe(III) ions, thus forming six μ_1,3_ bridges. The coordination sphere of each Fe(III) is completed by a methanol ligand and the central oxo atom.

The Fe…Fe interatomic distances are almost equal, being in the range of 3.266–3.310 Å (Table 3) with the three iron(III) ions arranged on the vertices of an almost isosceles triangle. The three Fe(III) ions are triply bridged by an oxo atom (O1) located in the center of the triangle, with Fe–O1 distances ranging between 1.863 (2) and 1.926 (2) Å (Table 3). The three Fe–O–Fe angles around the central O atom (O1) range between 119.04 (11)° and 121.40 (12)°, and their sum (=360°) indicates the co-planarity of the four atoms that make up the core [Fe_3_(µ_3_-O)] (Figure 4B). Among the bond distances around the Fe(III) ions, O_oxo_ is closest to the iron(III) ions (Fe–O1 = 1.863 (2)–1.905 (2) Å), and the methanol oxygen atoms are at the longest distances (Fe–O_methanol_ = 2.057 (2)–2.078 (2) Å), which are slightly longer than the Fe–O_carboxylato_ distances (2.002 (2)–2.064 (2) Å) (Table 3 and Table S5). In the anionic complex [FeCl_4_]^−^, the four-coordinated iron(III) ion Fe(4) is in an almost ideal tetrahedral geometry (Fe–Cl = 2.1483 (12)–2.1857 (12) Å, Cl–Fe_4_–Cl = 106.25 (5)–113.78 (5)°), having a similar arrangement to a few reported examples containing a tetrachloroferrate counter anion [98,99,100,101,102,103].

Intramolecular H-bonds are developed between the hydroxyl H of methanol ligands and carboxylate oxygen atoms of diclofenac ligands. The solvate water, methanol and Hdicl molecules are all interacting and stabilized in the structure by the development of intermolecular H-bonds (Table S4).

### 2.3. Spectroscopic Study of the Complexes

The spectroscopic characterization of the complexes focused on the attribution of the data derived by infrared, electronic (UV-vis), Mössbauer, and EPR spectroscopies.

The existence of the ligands and the binding mode of the NSAIDs in complexes **1**–**3** were studied using IR spectroscopy. In the IR spectra of the complexes (Figure S1), the band located at 1590–1596 cm^−1^ could be attributed to the antisymmetric, *ν*_asym_(COO)-stretching vibration of the carboxylato groups of the NSAIDs, and the band at 1422–1429 cm^−1^ could be assigned to the symmetric, *ν*_sym_(COO)-stretching vibration of the NSAIDs’ carboxylato groups. Their presence may indicate the deprotonation of the carboxylic group. In addition, the parameter Δ*ν*(COO) (=*ν*_asym_(COO) − *ν*_sym_(COO)) has values in the range 161–174 cm^−1^, which are lower than those presented for the corresponding salts, and may suggest a bidentate fashion [104,105], which is in good agreement with the crystal structures discussed. Additionally, the characteristic bands of the out-of-plane ρ(C–H) vibrations of the corresponding nitrogen donor co-ligand appear at 723 cm^−1^ for ρ(C–H)_phen_ in complex **1** and at 694 cm^−1^ for ρ(C–H)_py_ in **2**. The presence of these bands confirms the coordination of the nitrogen donors in these complexes [104].

The UV-vis spectra of the complexes were recorded both in the solution (i.e., in DMSO, as well as in the presence of the buffer solution used in the DNA/albumin interaction studies) and in a solid state (as nujol mull) in order to explore all possible changes upon dilution. The similarity of the spectra suggests that complexes **1**–**3** keep the same structure in the solution [22,46,51,52,63,73,75,76,77]. One band located at λ_max_ = 490–515 nm (ε = 400–580 M^−1^ cm^−1^) is observed in the visible region of the spectrum, which may be attributed to a d–d transition or a ^6^A_1g_ → ^5^T_1g_ or ^6^A_1g_ → T_2g_(G) transition [106], which are characteristic for distorted octahedral Fe^3+^ complexes [107,108]. For the oxo-bridged Fe(III) complexes **2** and **3**, the band located at 357–366 nm (ε = 6100–7200 M^−1^cm^−1^) may be attributed to a charge-transfer transition from the oxo-group to the Fe(III) ion. In addition, in the UV region of the spectrum, the intense bands appearing in the range λ = 285–297 nm (ε = 9660–14,000 M^−1^cm^−1^) may be attributed to intra-ligand transitions [109].

**Figure 5:**
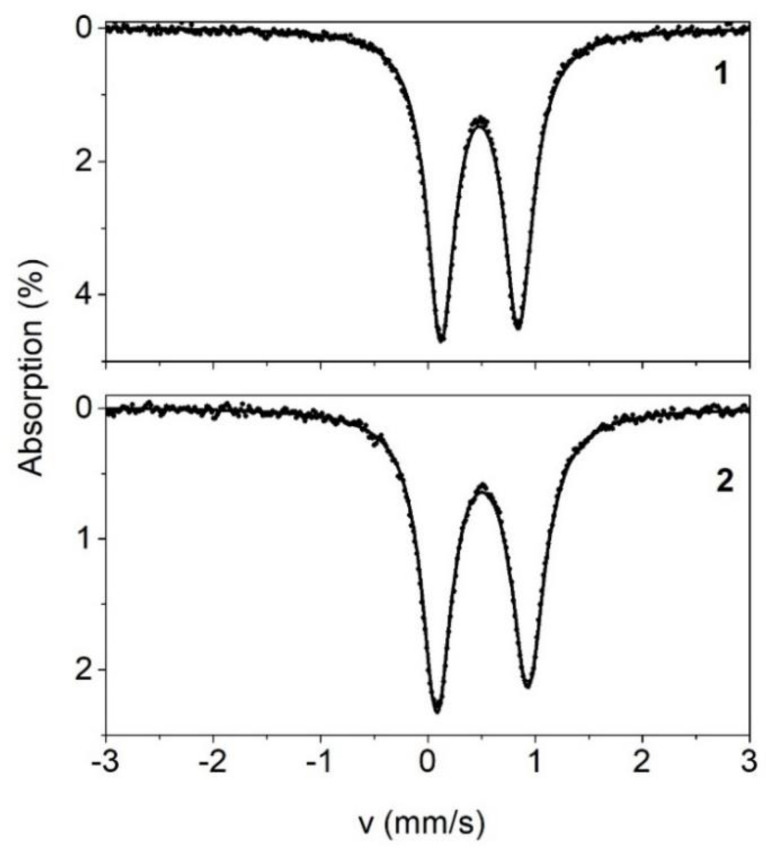
Mössbauer spectra from powder samples of **1** and **2** at 80 K. The black solid lines are theoretical simulations obtained with the parameters listed in Table 4.

The Mössbauer spectra from powder samples of complexes **1** and **2** recorded at 80 K are shown in Figure 5. The spectra comprise an asymmetric quadrupole doublet with parameters quoted in Table 4. The values of the isomer shift, δ, and the quadruple splitting parameter, ΔE_Q_, are consistent with Fe(III) (S = 5/2) ions with the ligand composition (O, N) of the iron sites in **1** and **2** [110]. The asymmetry of the doublets is attributed to relaxation effects [110].

**Figure 6:**
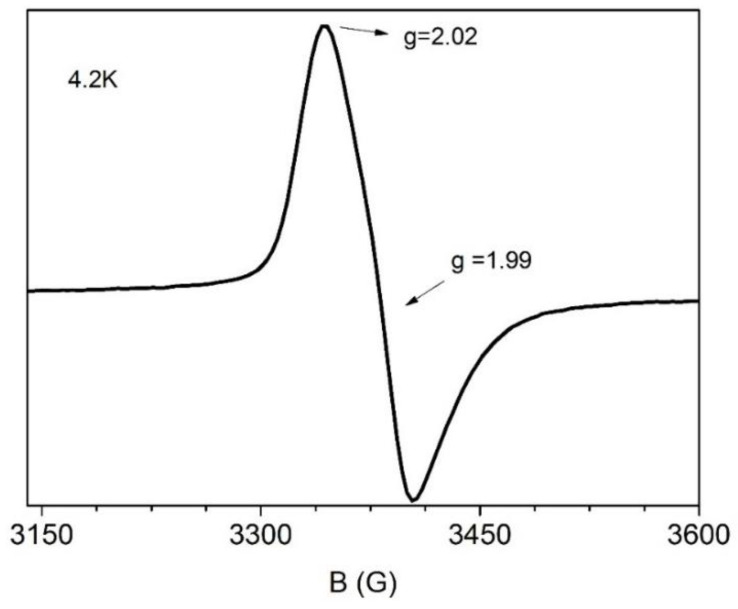
X-band EPR spectrum from a powder sample of **2** at 4.2 K. EPR conditions: modulation amplitude, 10 G_pp_, microwave power, 2 mW; microwave frequency, 9.42 GHz.

The X-band EPR spectrum of a solid powder sample of **2** recorded at 4.2 K is shown in Figure 6. The spectrum comprises a strong signal at g ~2.0, exhibiting a weak anisotropy. Such signals are often observed in antiferromagnetically coupled triferric clusters and are consistent with an S = 1/2 ground state [111,112].

### 2.4. Anticholinergic Activity of the Complexes

Metal ions, such as Cu, Fe, and Zn, in the brain are critical regarding the proper functioning of enzymes involved in neurotransmission and aging [113]. The compromise of metal ion homeostasis has been linked to various neurodegenerative diseases [113,114,115,116]. For instance, a high concentration of the biometal ions Cu, Fe, and Zn, as well as elevated oxidative stress levels, have been found in the brain of AD patients [113]. Promising compounds, such as metal-based drugs, have been proposed to act on different molecular targets and to contribute to the treatment of neurodegenerative diseases [116]. This fact, along with our previously reported results on metal complexes with anticholinergic activity [75,76], has led to the study of the anticholinergic activity of the synthesized ferric complexes.

Alzheimer’s disease is characterized by the growing damage of neural tissues in the brain. The neurotransmitter acetylcholine is responsible for maintaining communication between neurons in the brain [117]. Deficiency of this neurotransmitter is caused by the impaired activity of the enzyme AChE. The AChE inhibitors can enhance central cholinergic neurotransmission by preventing the degradation of acetylcholine [117,118].

As of now, the FDA (Food and Drug Administration, Silver Spring, MD, USA)-approved drugs for AD treatment include acetylcholinesterase inhibitors, or N-methyl-D-aspartate (NMDA)-receptor antagonists [113]. While these drugs only offer mild symptomatic relief in the memory of patients and improve neurotransmitter action over a period, they still are the most promising treatment for AD [117,118].

The two tested cholinesterases (AChE and BuChE) coexist, compensating for each other, in order to maintain the normal cholinergic pathways. AChE is the dominant cholinesterase in the human brain (healthy or early AD). In advanced AD, AChE levels are gradually reduced by 90% due to severe cholinergic neuronal damage. At the same time, BuChE compensates for the lack of AChE. Its levels and function increase to 105–165% of normal levels, making it the major metabolic enzyme of acetylcholine. It is important therefore to assess the inhibition of each cholinesterase for the different stages of AD [75,76].

In order to investigate the potency of the compounds, the rate of inhibition (I%) of each enzyme by the compounds (Table 5) was calculated at the standard concentration c = 10^−3^ M according to Equation (1) (Section 3.4.1). The selectivity index (SI) (defined as I_BuChE_/I_AChE_) was also calculated (Table 5). Neostigmine methyl sulfate (Neo) was used as reference compound [75,76].

Regarding BuChE inhibition, the activity of the tested compounds is significantly higher than that of the free NSAIDs sodium diclofenac and diflunisal, emphasizing the value of the coordination of the drugs to the iron ion. All complexes **1**–**3** showed significant activity against the enzyme, with complex **1** exhibiting the highest potency with 79.25% activity at a concentration of 10^−3^ M. On the contrary, for the inhibition of AChE, the complexes showed substantially lower activity than the corresponding NSAID, with diflunisal having the highest potency among the tested compounds with only 17.25% activity at a concentration of 10^−3^ M.

As it can be also demonstrated by the SI values, complexes **1**–**3** favor the inhibition of BuChE, and hence may be proved more effective in the treatment of late-stage AD [118].

### 2.5. Antioxidant Activity of the Complexes

Oxidative stress is defined as the imbalance between the production of reactive oxygen species (ROS) and the ability of a biological system to inactivate toxic molecules and repair the damage they cause. The generation of oxidative stress is due to either the increased production of oxygen free radicals or the deficiency of the various antioxidant cellular mechanisms [119]. Any substance capable of protecting or delaying the oxidation of other molecules is called an antioxidant. They are compounds that bind with the free radicals by giving up their own electrons and subsequently inactivate their ability to cause damage to biological molecules [120]. The role of antioxidants is to prevent damage to cellular components by neutralizing or scavenging free radicals, which may be the cause of various heart diseases, cancer, inflammation, aging, autoimmune diseases, Alzheimer, Parkinson, and more [121]. The potential treatment of these diseases is based on the elimination of free radicals and oxidative stress from an antioxidant agent [29].

Most of the reported NSAIDs may act as inhibitors of free radical production or free radical scavengers [120]. Such compounds showing antioxidant activity may play a crucial role in the treatment of inflammation and potentially lead to effective drugs [29,74,75]. The potential antioxidants of free NSAIDs sodium diclofenac, diflunisal, and their complexes **1**–**3,** were evaluated by investigating their ability to scavenge DPPH, hydroxyl, and ABTS radicals [122,123], and they were compared with the antioxidant agents nordihydroguaiaretic acid (NDGA), butylated hydroxytoluene (BHT), and 6-hydroxy-2,5,7,8-tetramethylchromane-2-carboxylic acid (trolox), which are among the most known reference compounds (Table 6) [29,74].

The DPPH-scavenging is usually related with potential protection against rheumatoid arthritis and inflammation and may be often involved in antiageing and anti-inflammatory treatment [124]. The DPPH radical-scavenging capacity was studied after treatment for two different time intervals (20 min and 60 min). The DPPH-scavenging activity of complexes **1**–**3** is time-independent, as no significant differences were observed after 20-min and 60-min treatments (Table 6). However, the complexes present a rather low ability towards DPPH radicals when compared to the reference compounds BHT and NDGA (Table 6), with complex **2** possessing slightly higher ability than the other complexes (DPPH% = 22.97–23.12%).

The scavenging of hydroxyl radicals is evidence of the scavenging of ROS, and subsequently, the hydroxyl-scavengers may act protectively [124]. The activity of the complexes to scavenge hydroxyl radicals is significantly high (Table 6), with even higher activity than the reference compound trolox, with complex **3** being the most active OH-scavenger (OH% = 94.31 ± 0.78%).

The scavenging of the cationic ABTS radicals is a marker of the total antioxidant activity [125]. Complexes **1**–**3** present similar ABTS-scavenging ability, which is significantly high and close to that of the reference compound trolox (Table 6).

In conclusion, the complexes are better radical scavengers than the corresponding free NSAIDs, suggesting that the binding of the NSAID to Fe(III) results in pronounced antioxidant ability. Such results are in accordance with several reports where the metal complexes of bioactive ligands were better radical scavengers than the corresponding free NSAIDs. In comparison with the reported Fe(III)-fenamato complexes, complex **1** is the most active DPPH-scavenger, while complex **3** has the best ABTS-scavenging activity among the Fe(III)-NSAID complexes [22]. The radical scavenging activity of the complexes seems to be selective (complexes **1**–**3** scavenge ABTS and hydroxyl radicals much better than DPPH) and is in the range reported for other metal-NSAID complexes [46,62,63,64,71,73,126].

### 2.6. Interaction of the Complexes with Albumins

Serum albumin (SA) is the most abundant protein in blood serum and among the most important in the circulatory system [79]. It is synthesized in the liver and released as a non-glycosylated protein into the circulation. It is related with the transportation of non-esterified fatty acids, various metabolites, drugs, organic substances, and metal complexes through the bloodstream toward their biological targets (cells and tissues) [80,81]. The binding to such proteins may lead to a loss or an increase in the biological properties of the original drug or provide new paths for activity [127]. The best method to study the binding of drugs to albumins is by fluorescence emission spectroscopy [128]. Various studies have reported that the pharmacological and pharmacokinetic properties of drugs may depend on their interaction with this key carrier plasma protein [129]. The solutions of both albumins, HSA and BSA, when excited at 295 nm, exhibit an intense fluorescence emission band at ~351 nm and ~343 nm, respectively, which may be attributed to the tryptophan residues, namely a tryptophan-214 in HSA, and two tryptophan residues at positions 134 and 212 for its homologue BSA [130].

**Figure 7:**
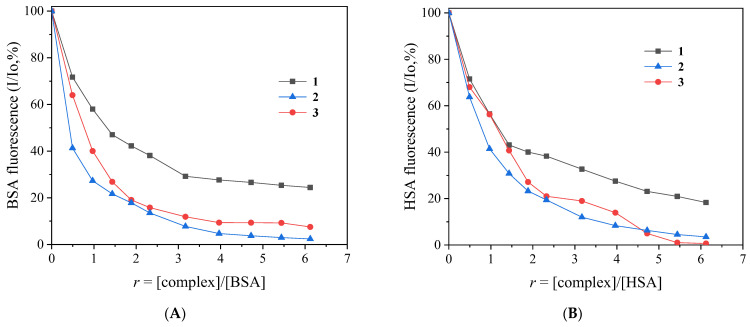
(**A**) Plot of % relative BSA fluorescence emission intensity (I/Io, %) at λ_em,max_ = 343 nm versus *r* (= [complex]/[BSA]) for complexes **1**–**3** (up to 24.4% of the initial BSA fluorescence for **1**, 7.5% for **2**, and 2.4% for **3**). (**B**) Plot of % relative HSA fluorescence emission intensity (I/Io %) at λ_em,max_ = 351 nm versus *r* (=[complex]/[HSA]) for complexes **1**–**3** (up to 18.3% of the initial HSA fluorescence for **1**, 0.6% for **2**, and 3.5% for **3**).

The addition of complexes **1**–**3** in the SA solution leads to an intense decrease in fluorescence intensity of up to 97.6% of for both albumins (Figure 7 and Table 7). Such quenching may indicate the binding of each complex to the albumin and may be assigned to changes in the tryptophan environment of SA due to possible changes in the protein’s secondary structure [130]. The inner-filter effect was checked with Equation (2) (Section 3.4.3) [131] and it was found to be negligible.

The Stern–Volmer constants (K_SV_) and the SA-quenching constants (K_q_) (Table 7) for the interaction of the complexes with SAs were determined using the Stern–Volmer equation (Equations (3) and (4), Section 3.4.3) and the corresponding plots (Figures S2 and S3). The values of K_q_ (Table 7) were relatively high, with complex **2** showing the highest values for both SAs (K_q(BSA)_ = 8.70 (±0.43) × 10^13^ M^−1^s^−1^ and K_q(HSA)_ = 6.09 (±0.30) × 10^13^ M^−1^s^−1^). The values of the quenching constants are much higher than 10^10^ M^−1^s^−1^, revealing the existence of a static quenching mechanism and indicating subsequently the interaction of the complexes with the albumins [127].

The SA-binding constant (K) of a compound must be high enough to infer tight binding for potential transportation and possible release. The values of K (Table 7) for the compounds were determined using the Scatchard equation (Equation (5), Section 3.4.3) and the corresponding plots (Figures S4 and S5). The K values were relatively high (of the magnitude 10^5^ M^−1^) and within the range reported for other Fe(III)- and metal-NSAID complexes [22]. These values satisfy the conditions of strong binding, safe transport, and the potential release at the target, as the values are quite lower than the binding constant of avidin with various compounds (of the 10^15^ M^−1^ order), which is among the strongest non-covalent interactions and may suggest a reversible binding to the albumin and subsequently the probability of release in the target cell [132].

### 2.7. Interaction of the Complexes with CT DNA

DNA is among the biomolecular targets for a series of drugs, as it is often involved in diverse mechanisms of action for such drugs [78], such as the inhibition of the nucleotide synthesis, inhibition of topoisomerase, and blockage of DNA replication [133]. Labile ligands in metal-based drugs (e.g., cisplatin) may offer vacant coordination sites for covalent binding to DNA bases [134], while non-labile and/or chelating ligands may provide stability to complexes and lead them to noncovalent interaction with DNA, i.e., via intercalation, groove-binding, and/or electrostatic interaction [134,135], as well as via chemical nuclease behavior [135]. The interaction of complexes **1**–**3** with CT DNA was studied by UV-vis spectroscopy, and viscosity measurements and via EB-competitive studies using fluorescence emission spectroscopy.

UV-vis spectroscopy is usually employed to obtain initial information regarding the interaction and the affinity between DNA and complexes, as revealed via the determination of the DNA-binding constant (K_b_). In the UV spectra of complexes **1**–**3**, the addition of incremental amounts of CT DNA induced slight changes to the intraligand bands of the spectra (Figure S6), i.e., slight hypochromism or hyperchromism of the bands located in the range 285–297 nm, which were accompanied by slight bathochromic shifts of the bands (Table 8). These features reveal the interaction of the complexes with CT DNA, which may lead to stabilization of the resulting complex-DNA adduct [136,137,138], although a conclusion of the interaction mode may not arise, making further experiments thus necessary, such as DNA viscosity measurements. The K_b_ values of complexes **1**–**3** are summarized in Table 8, as calculated using the Wolfe–Shimer equation (Equation (6), Section 3.4.4) [139] and the corresponding plots [DNA]/(ε_A_–ε_F_) versus [DNA] (Figure S7), and are lower than the classical intercalator EB (K_b_ = 1.23 (±0.07) × 10^5^ M^−1^) [140]. The K_b_ values of the complexes are similar to those reported for other metal-NSAID complexes, with complex **3** showing the highest DNA affinity among the complexes under study.

**Figure 8:**
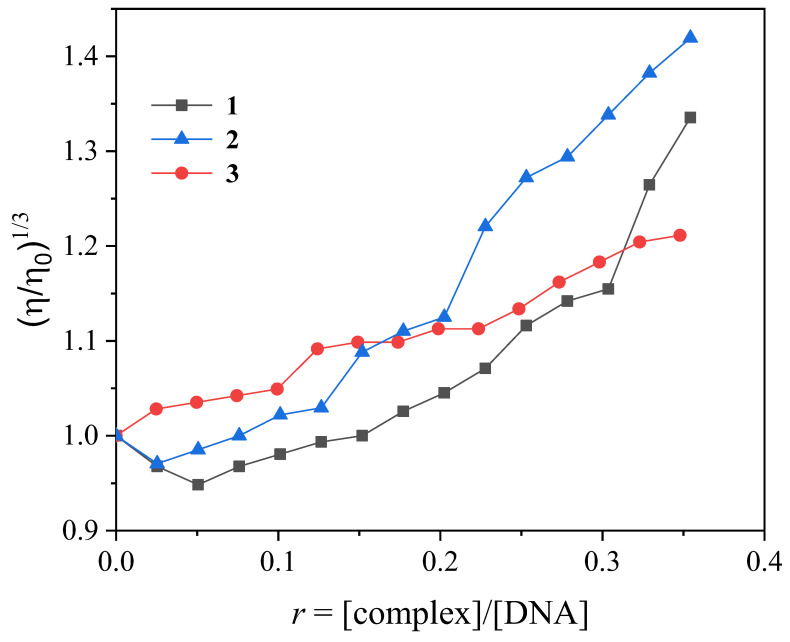
Relative viscosity of CT DNA (η/η_ο_)^1/3^ in buffer solution (150-mM NaCl and 15-mM trisodium citrate at pH 7.0) in the presence of complexes **1**–**3** at increasing amounts (*r* = [complex]/[DNA]).

The DNA viscosity measurement, as a hydrodynamic measurement, is a method to further investigate and clarify the interaction mode of compounds with CT DNA. In the intercalation model, the relative DNA viscosity will show an increase, while in the case of nonclassical intercalation (groove-binding or electrostatic interaction), the relative DNA viscosity will either decrease slightly or remain practically unchanged [141]. The viscosity of a CT DNA solution (0.1 mM) was monitored upon the addition of increasing amounts of complexes **1**–**3** and presented an increase upon the gradual addition of the complexes (Figure 8). This increase in DNA viscosity may serve as evidence of an intercalative interaction between DNA and each complex that results in longer separation distances between DNA bases upon the insertion of the complexes in-between the DNA bases [142,143].

**Figure 9:**
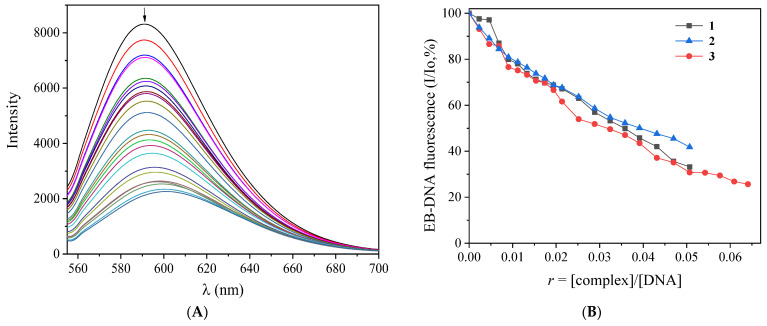
(**A**) Fluorescence emission spectra (λ_ex_ = 540 nm) of EB–DNA ([EB] = 20 μM, [DNA] = 26 μM) in buffer solution (150-mM NaCl and 15-mM trisodium citrate at pH7.0) in the absence and presence of increasing amounts of complex **3** (different colors). The arrow shows the changes in intensity upon increasing amounts of **3**. (**B**) Plot of relative EB–DNA fluorescence intensity (I/Io, %) at λ_em_ = 592 nm versus *r (r* = [complex]/[DNA]) in the presence of complexes **1**–**3** (up to 33.2% of the initial EB–DNA fluorescence for **1**, 41.8% for **2**, and 25.7% for **3**).

Ethidium bromide is a known fluorescent compound which intercalates into DNA bases. The competition of a compound with EB is a usual means to confirm the DNA-binding mode. The solutions of NSAIDs and their complexes do not fluoresce either alone or in the presence of the CT DNA or EB solution at room temperature when excited at 540 nm. Therefore, the changes occurring in the fluorescence emission spectra of an EB–DNA solution upon addition of the compounds may be used to study the EB-displacing ability of the compounds from the EB–DNA adduct [130]. The EB–DNA adduct was prepared after a 1-h pretreatment of a solution containing EB ([EB] = 20 μM) and CT DNA ([DNA] = 26 μM). The fluorescence emission spectra of this solution were recorded in the presence of increasing amounts of complexes **1**–**3** (shown for complex **3** in Figure 9A). The addition of the complexes resulted in a quenching of the EB–DNA emission band at 592 nm (up to ~74% of the initial EB–DNA fluorescence (Figure 9B and Table 9). Such quenching probably originated from the displacement of EB by the compounds, revealing their competition with complexes for the DNA intercalation sites [144].

The Stern–Volmer and quenching constants were determined with the corresponding plots (Figure S8) and Equations (3) and (4) (Section 3.4.3) [130]. These constants are in the range reported for other metal-NSAID complexes [46,51,62,63,64,71,73,126], with complex **3** presenting the highest constants among the complexes studied herein. The values of K_q_ (Table 9) are much higher than the value of 10^10^ M^−1^s^−1^, suggesting a static quenching mechanism because of the formation of a new adduct, obviously between the DNA and the complex [145].

## 3. Materials and Methods

### 3.1. Materials–Instrumentation–Physical Measurements

The chemicals reagents, FeCl_3_∙6H_2_O, py, phen, CT DNA, BSA, HSA, EB, DPPH, ABTS, EDTA, BHT, NDGA, and trolox were purchased from Sigma-Aldrich Co. (St. Louis, MO, USA). NaCl, KOH and trisodium citrate were purchased from Merck (Rahway, NJ, USA). Sodium diclofenac was purchased from Tokyo Chemical Industry and diflunisal from Fluka (Buchs, Switzerland). The reagents for the evaluation of the cholinergic activity: 5,5-dithio-bis-(2-nitrobenzoic acid) (DTNB), electric eel acetylcholinesterase (eeAChE), acetylthiocholine iodide (ATCI), equine serum butyrylcholinesterase (eqBuChE), S-butyrylthiocholine iodide (BTCI), and neostigmine methyl sulfate (Neo) were purchased from J&K Scientific Co. (Beijing, China). Ascorbic acid, Na_2_HPO_4_, and NaH_2_PO_4_ were purchased from Chemlab Co. (Zedelgem, Belgium). All the chemical reagents and all solvents were of reagent grade and were used as purchased from commercial sources.

The stock solution of CT DNA was prepared via dilution of CT DNA to a buffer solution (containing 150-mM NaCl and 15-mM trisodium citrate at pH 7.0) followed by exhaustive stirring and it was kept at 4 °C for no longer than two weeks. This stock solution gave a ratio of UV absorbance at 260 and 280 nm (A_260_/A_280_) in the range of 1.85–1.90, an indication that DNA was sufficiently free of protein contamination [146]. The DNA concentration was determined by the UV absorbance at 260 nm after 1:20 dilution using ε = 6600 M^−1^cm^−1^ [147].

Infrared (IR) spectra were recorded in the range (400–4000 cm^−1^) on a Nicolet FT-IR 6700 spectrometer (Thermo Fisher Scientific, Waltham, MA, USA) with samples prepared as KBr pellets (abbreviations used: vs. = very strong; s = strong; m = medium; Δ*ν*(COO) = *ν*_asym_(COO) − *ν*_sym_(COO)). UV-visible (UV-vis) spectra were recorded as nujol mulls and in solution (in the concentration range of 10^−5^ to 10^−3^ M) on a Hitachi U-2001 dual-beam spectrophotometer (Hitachi High-Tech Corporation, Ibaraki, Japan). C, H, and N elemental analyses were performed on a PerkinElmer 240B elemental analyzer (PerkinElmer, Waltham, MA, USA). The molar conductivity measurements were carried out in a 1-mM DMSO solution of the complexes with a Crison Basic 30 conductometer (Crison Instruments, Barcelona, Spain). The fluorescence emission spectra were recorded in solution on a Hitachi F-7000 fluorescence spectrophotometer (Hitachi High-Tech Corporation, Ibaraki, Japan). The viscosity experiments were conducted using an ALPHA L Fungilab rotational viscometer (Fungilab S.A., Barcelona, Spain) equipped with an 18-mL LCP spindle.

The Mössbauer spectra from powdered samples were recorded with a constant acceleration conventional spectrometer with ^57^Co (Rh matrix) γ-ray source using a Janis cryostat. Isomer shifts were reported relative to α-Fe at room temperature. The spectra were analyzed using the program WMOSS (Web Research, Edina, MN, USA). X-band EPR measurements from powdered sample of **2** were carried out on an upgraded Bruker ER-200D spectrometer (Bruker, Athens, Greece) equipped with an Oxford ESR 9000 cryostat, an Anritsu MF76A frequency counter, and a Bruker 035 NMR Gaussmeter with the perpendicular mode standard cavity 4102ST.

### 3.2. Synthesis of the Complexes

#### 3.2.1. Synthesis of [Fe_2_(difl)_2_(MeO)_2_(phen)_2_]∙H_2_O, **1**

A methanolic solution (10 mL) containing H_2_difl (0.2 mmol, 50 mg) and KOH (0.4 mmol, 22 mg) was stirred for 1 h. The resultant solution was added simultaneously with a methanolic solution (5 mL) of phen (0.2 mmol, 36 mg) to a methanolic solution (10 mL) of FeCl_3_∙6H_2_O (0.2 mmol, 54 mg). After 2 days, red-brown crystals of [Fe_2_(difl)_2_(MeO)_2_(phen)_2_]∙H_2_O suitable for X-ray structure determination was deposited (yield: 55 mg, 52%). Anal. Calcd. for [Fe_2_(difl)_2_(MeO)_2_(phen)_2_]∙H_2_O (C_52_H_36_F_4_Fe_2_N_4_O_9_) (MW = 1048.56): C, 59.56; H, 3.46; N, 5.34%; found: C, 59.75; H, 3.55; N, 5.48%. IR spectrum (KBr disk), ν_max_/cm^−1^: *ν*_asym_(COO): 1590 (s); *ν*_sym_(COO): 1429 (s); Δ*ν*(COO) = 161; ρ(C–H)_phen_: 724 (m). UV-vis spectra: as nujol mull, λ/nm: 519; in DMSO, λ/nm (ε/M^−1^cm^−1^): 297 (shoulder (sh)) (14,000), 515 (500). Soluble in DMF and DMSO (Λ_M_ = 5 S·cm^2^·mol^−1^, in 1 mM DMSO solution).

#### 3.2.2. Synthesis of [Fe_3_O(dicl)_6_(py)_3_]Cl∙py, **2**

Complex **2** was prepared by the addition of a methanolic solution (10 mL) of Nadicl (0.4 mmol, 92 mg) to a methanolic solution (10 mL) of FeCl_3_∙6H_2_O (0.2 mmol, 54 mg), followed by the addition of 3 mL of py. The resultant solution was stirred for 30 min and was left to evaporate slowly. Brown crystals of [Fe_3_O(dicl)_6_(py)_3_]Cl∙py suitable for X-ray structure determination were deposited after 40 days (yield: 60 mg, 40%). Anal. and Calcd. for [Fe_3_O(dicl)_6_(py)_3_]Cl∙py, **2**, (C_104_H_80_Cl_13_Fe_3_N_10_O_13_) (MW = 2287.00): C, 54.16; H, 3.50; N, 6.07%; found: C, 53.98; H, 3.59; N, 5.99%. IR spectrum (KBr disk), ν_max_/cm^−1^: *ν*_asym_(COO): 1596 (vs); *ν*_sym_(COO): 1422 (s); Δ*ν*(COO) = 174; ρ(C–H)_py_: 694 (m). UV-vis spectra: as nujol mull, λ/nm: 363, 495 (sh); in DMSO, λ/nm (ε/M^−1^cm^−1^): 285 (12,500), 366 (7200), 490 (sh) (580). Soluble in DMSO (Λ_M_ = 65 S·cm^2^·mol^−1^, in 1-mM DMSO solution).

#### 3.2.3. Synthesis of [Fe_3_O(dicl)_6_(MeOH)_3_][FeCl_4_]·Hdicl·1.5MeOH∙H_2_O, **3**

The complex was prepared in a similar way to **2** in the absence of pyridine. Light-brown crystals of [Fe_3_O(dicl)_6_(MeOH)_3_][FeCl_4_]·Hdicl·1.5MeOH∙H_2_O (yield: 45 mg, 35%) suitable for X-ray structure determination were deposited after 10 days. Anal. And Calcd. for [Fe_3_O(dicl)_6_(MeOH)_3_][FeCl_4_]·Hdicl·1.5MeOH∙H_2_O (C_102.5_H_91_Cl_18_Fe_4_N_7_O_20.5_) (MW = 2610.43): C, 47.16; H, 3.51; N, 3.76%; found: C, 47.33; H, 3.43; N, 3.63%. IR spectrum (KBr disk), ν_max_/cm^−1^: *ν*_asym_(COO): 1592 (vs); *ν*_sym_(COO): 1422 (s); Δ*ν*(COO) = 170. UV-vis spectra: as nujol mull, λ/nm: 353, 511; in DMSO, λ/nm (ε/M^−1^cm^−1^): 288 (9660), 357 (6100), 515 (400). Soluble in DMSO (Λ_M_ = 70 S·cm^2^·mol^−1^, in 1-mM DMSO solution).

### 3.3. Single-Crystal X-ray Crystallography

Single crystals of complexes **1**–**3** suitable for crystal structure analysis were mounted at room temperature on a Bruker Kappa APEX2 diffractometer equipped with a Triumph monochromator using Mo Kα (λ = 0.71073 Å, source operating at 50 kV and 30 mA) radiation. Unit cell dimensions were determined and refined by using the angular settings of at least 200 high-intensity reflections (>10σ(I)) in the range of 11 < 2θ < 36°. Intensity data were recorded using φ and ω scans. All crystals presented no decay during the data collection. The frames collected for each crystal were integrated with the Bruker SAINT Software package [148] using a narrow-frame algorithm. Data were corrected for absorption using the numerical method (SADABS) based on crystal dimensions [149]. All structures were solved using SUPERFLIP [150] incorporated in Crystals. Data refinement (full-matrix least-squares methods on *F*^2^) and all subsequent calculations were carried out using the Crystals version 14.61 build 6236 program package [151]. All non-hydrogen, non-disordered atoms were refined anisotropically. For the disordered atoms, their occupation factors under fixed isotropic thermal parameters were first detected. Afterwards, all were refined with fixed occupation factors, isotropically in the case of compound **2** (pyridine solvate molecules and chlorido counter anions) and both anisotropically and isotropically in the case of compound **3** (anisotropically in the case of the non-coordinated diclofenac acid molecules and isotropically in the case of methanol and water solvate molecules).

Hydrogen atoms riding on non-disordered parent atoms were located from difference Fourier maps and refined at idealized positions riding on the parent atoms with isotropic displacement parameters Uiso(H) = 1.2Ueq(C) or 1.5Ueq(methyl, –NH and –OH hydrogens) and at distances C–H 0.95 Å, N–H 0.83 Å, and O–H 0.82 Å. All methyl, amine, and OH hydrogen atoms were allowed to rotate but not to tip. Hydrogen atoms riding on disordered oxygen atoms of methanol and water solvent molecules were positioned geometrically to fulfill hydrogen bonding demands. The rest of the methyl and aromatic hydrogen atoms were positioned geometrically to their parent atoms. The crystallographic data for complexes **1**–**3** are presented in Table S1. Further details on the crystallographic studies as well as atomic displacement parameters are given as Supplementary Materials in the form of CIF files.

### 3.4. Evaluation of the Biological Profile

In order to study in vitro the biological activity of complexes **1**–**3** (i.e., anticholinergic activity, free radical scavenging, and interaction with CT DNA and serum albumins), they were dissolved in DMSO (1-mM) due to their low aqueous solubility. The mixing of each solution with the aqueous buffer solution of DNA or albumins used in the studies never exceeded 5% DMSO (*v*/*v*) in the final solution.

#### 3.4.1. Anticholinergic Activity

In the study of cholinesterase inhibitors, the inhibitory effect of the compounds (the NSAIDs and their complexes **1**–**3**) against AChE and BuChE was examined using a modified methodology based on Ellman’s method [152,153].

The ability of the compounds to inhibit AChE and BuChE was evaluated using UV-vis spectroscopy. All the assays were carried out in a 0.1M NaH_2_PO_4_/Na_2_HPO_4_ buffer at a pH = 7.4. Enzyme solutions were prepared with 2.0 U/mL for AChE and 3.0 U/mL for BuChE. A reaction mixture containing 20 μL of phosphate buffer, 100 μL of DTNB (1-mM), and 40 μL of the enzyme (AChE and BuChE) was incubated with 20 μL of the compounds at various concentrations at 37 °C for 15 min. The reaction was started by the addition of the substrate (20-μL) of ATCI or BTCI solution (1-mM), respectively, and incubation for additional 3 min.

The enzyme activity was determined by measuring the increase in absorbance at 2-min intervals at 412 nm at 37 °C (ε = 14,150 M^−1^cm^−1^). The anticholinergic activity of the compounds was expressed as the percent inhibition of AChE and BuChE at the standard concentration c = 10^−3^ M.

The rate of inhibition of each enzyme by the compounds was calculated according to the following expression:

(1) $$I\%=\left(1-\frac{V_{B}}{V_{A}}\right)\times 100$$

where V_B_ and V_A_ indicate the absorbance measured for ChEs in the presence and absence of inhibitors, respectively. The results are expressed as the average of three repetitions of the tests performed and the standard deviation was less than 3% of the mean [154]. The selectivity index (SI), defined as IC_50_ BuChE/IC_50_ AChE, was also calculated. Neostigmine methyl sulfate was used as the appropriate standard.

#### 3.4.2. Antioxidant Activity

The antioxidant activity of complexes **1**–**3** was evaluated via their ability to scavenge the free radicals DPPH, hydroxyl, and ABTS. Each experiment was performed in triplicate and the standard deviation of absorbance was less than 10% of the mean.

**Determination of the reducing activity of the stable radical DPPH:** To an ethanolic solution of DPPH (0.1 mM) was added an equal volume of an ethanolic solution of complexes **1**–**3**, which had a concentration of 0.1 mM. Ethanol was also used as a control solution. The absorbance at 517 nm was recorded at room temperature, two times, after 20 and 60 min, in order to examine the time-dependence of the DPPH-scavenging activity [124]. The DPPH-scavenging activity of the compounds was expressed as the percentage reduction of the absorbance values of the initial DPPH solution (RA%). The compounds NDGA and BHT were used as reference compounds.

**Competition of the tested compounds with DMSO for hydroxyl radicals:** The hydroxyl radicals generated by the Fe^3+^/ascorbic acid system were detected according to Nash by the determination of formaldehyde produced from the oxidation of DMSO [155]. The reaction mixture contained EDTA (0.1 mM), Fe^3+^ (167 μM), DMSO (33 mM) in phosphate buffer (50 mM, pH 7.4), complexes **1**–**3** (concentration 0.1 mM), and ascorbic acid (10 mM). After a 30-min incubation at 37 °C, the reaction was stopped with CCl_3_COOH (17% *w*/*v*) and the absorbance at λ = 412 nm was measured. Trolox was used as a reference compound. The competition of the compounds with DMSO for •OH, generated by the Fe^3+^/ascorbic acid system, expressed as the percentage of inhibition of formaldehyde production, was used for the evaluation of their hydroxyl radical-scavenging activity (OH%).

**Assay of radical cation scavenging activity (ABTS^+•^):** An ABTS cationic radical (ABTS^+•^) was produced by reacting an aqueous stock solution (2 mM) of ABTS with 0.17-mM potassium persulfate and allowing the mixture to stand in the dark at room temperature for 12–16 h before use. Because ABTS and potassium persulfate react stoichiometrically at a ratio of 1:0.5, this results in the incomplete oxidation of the ABTS. Although the oxidation of ABTS commenced immediately, the absorbance became maximal and stable after 6 h. The radical was stable in this form for more than two days when stored in the dark at room temperature. The ABTS radical solution was diluted with ethanol to an absorbance of 0.70 at 734 nm. After the addition of 10 μL of complexes **1**–**3** or the standards (0.1 mM) in DMSO, the absorbance was recorded exactly 1 min after initial mixing [124]. The radical scavenging activity of the complexes was expressed as the percentage inhibition of the absorbance of the initial ABTS solution (ABTS%). Trolox was used as a reference compound.

#### 3.4.3. Interaction with Serum Albumins

The albumin-binding study for complexes **1**–**3** was performed via fluorescence emission quenching experiments using BSA (3 μM) or HSA (3 μM), respectively, in a buffer solution (containing 15-mM trisodium citrate and 1500 mM NaCl at pH 7.0). The quenching of the emission intensity of tryptophan residues of BSA at 343 nm or HSA at 351 nm was monitored using complexes **1**–**3** as quenchers with increasing concentrations [130]. The fluorescence emission spectra were recorded in the range of 300–500 nm, with an excitation wavelength of 295 nm. The quantitative studies of the serum albumin fluorescence spectra were performed after correction by subtracting the spectra of the compounds.

The extent of the inner-filter effect can be roughly estimated with the following formula:

(2) $$I_{\mathrm{corr}}=I_{\mathrm{meas}}\times 10^{\frac{\varepsilon \left(\lambda_{\mathrm{exc}}\right)\mathrm{cd}}{2}}\times 10^{\frac{\varepsilon \left(\lambda_{\mathrm{em}}\right)\mathrm{cd}}{2}}$$

where I_corr_ = corrected intensity, I_meas_ = the measured intensity, c = the concentration of the quencher, d = the cuvette (1 cm), ε(λ_exc_), and ε(λ_em_) = the ε of the quencher at the excitation and the emission wavelength, respectively, as calculated from the UV-vis spectra of the complexes [131].

The Stern–Volmer and Scatchard graphs are used in order to study the interaction of a quencher with serum albumins [130]. The Stern–Volmer quenching equation is used [130]:

(3) $$\frac{\mathrm{Io}}{I}=1+K_{q}\times \tau_{0}\times \left[Q\right]=1+K_{\mathrm{SV}}\times \left[Q\right]$$

where Io = the initial tryptophan fluorescence intensity of SA, I = the tryptophan fluorescence intensity of SA after the addition of the quencher, K_q_ = the quenching constants of SA, K_SV_ = the Stern–Volmer constant, τ_o_ = the average lifetime of SA without the quencher, and [Q] = the concentration of the quencher. The value of K_SV_ (M^−1^) can be obtained by the slope of the diagram Io/I versus [Q]. Taking τ_o_ = 10^−8^ s as the fluorescence lifetime of tryptophan in SA, the value of K_q_ (M^−1^s^−1^) is calculated from the equation:

(4) $$K_{\mathrm{SV}}=K_{q}\times \tau_{o}$$

and from the Scatchard equation [130]:

(5) ΔIIo[Q]=n×K−K×ΔIIo

where n is the number of binding sites per albumin and K is the SA-binding constant, K (in M^−1^) is calculated from the slope in plots (ΔI/Io)/[Q] versus (ΔI/Io), and n is given by the ratio of y-intercept to the slope [130].

#### 3.4.4. Interaction with CT DNA

The interaction of the complexes with CT DNA was investigated by UV-vis spectroscopy and viscosity measurements and via the evaluation of their EB-displacing ability studied by fluorescence emission spectroscopy.

**Binding study with CT DNA by UV-vis spectroscopy:** The interaction of complexes **1**–**3** with CT–DNA was studied by UV-vis spectroscopy in order to investigate the possible binding modes to CT DNA and to calculate the DNA-binding constants (K_b_). Control experiments with DMSO were performed, and no changes in the spectra of CT DNA were observed. The value of K_b_ can be obtained by monitoring the changes in the absorbance at the corresponding λmax in the UV-vis spectra of each complex (10–30 μM), recorded with increasing concentrations of CT DNA (diverse *r* values) and given by the ratio of the slope to the y intercepts in the plots [DNA]/(εA–εf) versus [DNA], according to the Wolfe–Shimer equation [139]:

(6) $$\frac{\left[\mathrm{DNA}\right]}{\left(\varepsilon_{A}-\varepsilon_{f}\right)}=\frac{\left[\mathrm{DNA}\right]}{\left(\varepsilon_{b}-\varepsilon_{f}\right)}+\frac{1}{K_{b}\times \left(\varepsilon_{b}-\varepsilon_{f}\right)}$$

where [DNA] is the concentration of DNA in base pairs, ε_A_ = A_obsd_/[compound], ε_f_ = the extinction coefficient for the free compound, and ε_b_ = the extinction coefficient for the compound in the fully bound form.

**CT DNA-binding studies using viscosity measurements:** The viscosity of DNA ([DNA] = 0.1 mM) in the buffer solution (150 mM NaCl and 15 mM trisodium citrate at pH 7.0) was measured in the presence of increasing amounts of complexes **1**–**3** (up to the value *r* = 0.36). All measurements were performed at room temperature. The obtained data are presented as (η/η_0_)^1/3^ versus *r*, where η is the viscosity of DNA in the presence of the compound, and η_0_ is the viscosity of DNA alone in buffer solution.

**EB-displacement studies:** The competition of complexes **1**–**3** with EB was investigated by fluorescence emission spectroscopy in order to examine whether the complexes can displace EB from its DNA–EB conjugate. The DNA–EB conjugate was prepared by adding 20-μM EB and 26-μM CT DNA in the buffer solution (150-mM NaCl and 15-mM trisodium citrate at pH 7.0). The possible intercalating effect of the complexes was studied by the addition of a certain amount of a solution of the compound into a solution of the DNA–EB conjugate. The influence of each compound to the DNA–EB complex solution was obtained by recording the changes in the fluorescence emission spectra with the excitation wavelength (λ_ex_) at 540 nm [130]. Complexes **1**–**3** do not show any appreciable fluorescence emission bands at room temperature in the solution or in the presence of CT DNA or EB under the same experimental conditions (λ_ex_ = 540 nm); therefore, the observed quenching of the EB–DNA solution may be attributed to the displacement of EB from its EB–DNA conjugate.

The Stern–Volmer constant K_SV_ is used to evaluate the quenching efficiency for each compound according to the Stern–Volmer equation (Equation (3)) [130], where Io and I are the emission intensities in the absence and the presence of the quencher, respectively, and [Q] is the concentration of the quencher (i.e., complexes **1**–**3**). The value of K_SV_ is obtained from the Stern–Volmer plots by using the slope of the diagram Io/I versus [Q]. Taking τ_o_ = 23 ns as the fluorescence lifetime of the EB–DNA system [144], the EB–DNA quenching constants (K_q_, in M^−1^s^−1^) of the compounds can be determined according to Equation (4).

## 4. Conclusions

Three novel Fe(III) complexes with the NSAIDs diflunisal and diclofenac have been isolated and their structural and spectroscopic features have been discussed. In the dinuclear centrosymmetric complex [Fe_2_(difl)_2_(MeO)_2_(phen)_2_]∙H_2_O (complex **1**), the iron(III) ions are bridged by two methoxo groups. Both complexes [Fe_3_O(dicl)_6_(py)_3_]Cl∙py (complex **2**) and [Fe_3_O(dicl)_6_(MeOH)_3_][FeCl_4_]·Hdicl·1.5MeOH∙H_2_O (complex **3**) contain a trinuclear cationic oxo-centered carboxylate-bridged complex of the “basic” carboxylates family which is neutralized by a chlorido or tetrachloroferrate counter anion, respectively. Due to the low aqueous solubility of complexes **1**–**3**, the studies in relation to the solution used were mainly performed using DMSO solutions of the complexes.

The complexes exhibited in vitro showed significant affinity for the albumins BSA and HSA, and they may bind tightly and reversibly to both SAs. The most possible binding mode of the complexes to CT DNA is via intercalation in-between DNA bases, and their binding is tight.

The investigation of the in vitro scavenging activity of complexes **1**–**3** towards DPPH, ABTS, and hydroxyl radicals revealed that the complexes are more active than the corresponding free NSAIDs and present selective activity towards hydroxyl and ABTS radicals versus DPPH radicals.

The anticholinergic activity of the complexes revealed that the Fe(III)-NSAID complexes **1**–**3** show better activity than the free NSAIDs sodium diclofenac and diflunisal against BuChE. As also established by the selectivity index, complexes **1**–**3** appear more potent for the late stages of AD. It should be noted that the results of this study have offered encouraging information about the potency of novel Fe(III)-NSAID complexes as anti-dementia agents. Thanks to this, future efforts could aim at figuring out potential applications of the studied complexes or evaluating the anticholinergic activity of other metal-NSAID complexes.

In conclusion, the results of the present study revealed a promising synergism of the NSAIDs diflunisal and sodium diclofenac with the bioelement iron and may initiate more elaborate biological studies and potential biological applications. A combination of the beneficiary effectiveness of the coordination compounds towards cholinesterase enzymes and their noteworthy radical-scavenging capacity may trigger the investigation of reported antioxidants as potential candidate anti-dementia agents.

## Figures and Tables

**Table 1:** Selected structural features (bond lengths (Å) and angles (°)) for complex **1**.

**Bond**	**Length (Å)**	**Bond**	**Length (Å)**
Fe1–O4 ^i^	1.9984 (19)	Fe1–N1	2.200 (2)
Fe1–O1	1.920 (2)	Fe1–N2	2.191 (3)
Fe1–O4	1.9726 (19)	Fe1–O3	1.928 (2)
Fe1…Fe1 ^i^	3.119		
**Bonds**	**Angle (°)**	**Bonds**	**Angle (°)**
O4^i^–Fe1–O1	172.52 (8)	N1–Fe1–O4	164.56 (9)
N2–Fe1–O3	163.00 (8)	Fe1 ^i^–O4–Fe1	103.54 (9)
O4^i^–Fe1–O4	76.46 (9)		

Symmetry code: (^i^) −x + 1, −y + 1, −z + 1.

**Table 2:** Selected structural features (bond lengths (Å) and bond angles (°)) for complex **2**.

**Bond**	**Length (Å)**	**Bond**	**Length (Å)**
Fe1–O_carboxylato_	2.018 (3)–2.029 (2)	Fe3–O_carboxylato_	2.021 (3)–2.041 (3)
Fe1–O13	1.910 (2)	Fe3–O13	1.890 (2)
Fe1–N1	2.157 (3)	Fe3–N3	2.179 (3)
Fe2–O_carboxylato_	1.995 (3)–2.039 (3)	Fe1…Fe2	3.311
Fe2–O13	1.922 (2)	Fe1…Fe3	3.296
Fe2–N2	2.149 (3)	Fe2…Fe3	3.302
**Bonds**	**Angle (°)**	**Bonds**	**Angle (°)**
O3–Fe1–O7	171.83 (11)	O4–Fe3–O5	170.17 (10)
O1–Fe1–O9	168.85 (11)	O10–Fe3–O12	170.68 (11)
O13–Fe1–N1	176.26 (12)	O13–Fe3–N3	176.44 (12)
O2–Fe2–O6	170.94 (11)	Fe2–O13–Fe1	119.54 (12)
O8–Fe2–O11	172.72 (11)	Fe1–O13–Fe3	120.29 (13)
O13–Fe2–N2	177.70 (12)	Fe2–O13–Fe3	120.05 (13)

**Table 3:** Selected structural features (bond lengths (Å) and bond angles (°)) for complex **3**.

**Bond**	**Length (Å)**	**Bond**	**Length (Å)**
Fe1–O1	1.863 (2)	Fe3–O1	1.905 (2)
Fe1–O_carboxylato_	2.002 (2)–2.051 (2)	Fe_3_–O_carboxylato_	2.024 (2)–2.042 (2)
Fe1–O8	2.057 (2)	Fe3–O10	2.064 (2)
Fe2–O1	1.926 (2)	Fe1…Fe2	3.266
Fe2–O_carboxylato_	2.026 (2)–2.060 (2)	Fe1…Fe3	3.287
Fe2–O9	2.078 (2)	Fe2…Fe3	3.310
Fe4–Cl	2.1483 (12)–2.1857 (12)		
**Bonds**	**Angle (°)**	**Bonds**	**Angle (°)**
O1–Fe1–O8	177.10 (9)	O1–Fe3–O10	176.72 (10)
O2–Fe1–O13	173.90 (9)	O14–Fe3–O15	172.32 (9)
O4–Fe1–O11	167.34 (9)	O5–Fe3–O6	171.38 (9)
O1–Fe2–O9	179.15 (9)	Fe2–O1–Fe3	119.54 (11)
O12–Fe2–O16	171.74 (10)	Fe3–O1–Fe1	121.40 (12)
O3–Fe2–O7	173.37 (10)	Fe2–O1–Fe1	119.04 (11)
Cl–Fe4–Cl	106.25 (5)–113.78 (5)		

**Table 4:** Mössbauer parameters at 80 K for the complexes **1**–**3**.

Complex	δ (mm/s) ^a^	ΔΕ_Q_ (mm/s) ^b^	Γ_L_ (mm/s) ^c,d^	Γ_R_ (mm/s) ^c,d^
1	0.48	0.72	0.32	0.33
2	0.51	0.85	0.34	0.38

^a^ ±0.01 mm/s; ^b^ ±0.02 mm/s; ^c^ full width at half maximum; ^d^ ±0.02 mm/s.

**Table 5:** Inhibition rate (%) of cholinesterases AChE and BuChE at 10^−3^ M of the compounds and selectivity index (SI = I_BuChE_/I_AChE_). Neostigmine methyl sulfate is the reference compound.

Compound	AChE (I%)	BuChE (I%)	SI
Complex **1**	1.77 ± 0.82	79.25 ± 2.00	44.77
Complex **2**	3.56 ± 0.11	74.36 ± 1.40	20.89
Complex **3**	3.55 ± 1.26	76.65 ± 1.95	21.59
Nadicl	10.31 ± 1.75	14.10 ± 0.12	1.37
H_2_difl	17.25 ± 1.46	22.87 ± 1.71	1.32
Neo ^a^	96.98 ± 0.02	98.38 ± 1.55	1.01

^a^ Neo = Neostigmine methyl sulfate and is used as reference compound.

**Table 6:** % DPPH scavenging ability (DPPH%), % ABTS radical scavenging activity (ABTS%), competition % with DMSO for hydroxyl radical (OH%) for diflunisal, sodium diclofenac, and their complexes **1**–**3**.

Compound	DPPH%, 20 min/60 min	OH%	ABTS%
H_2_difl	10.42 ± 0.56/14.31 ± 0.45	86.06 ± 0.38	76.58 ± 0.74
Complex **1**	12.57 ± 0.29/15.86 ± 0.62	91.08 ± 1.81	85.38 ± 0.92
Nadicl	18.26 ± 0.60/17.43 ± 0.23	75.46 ± 0.44	76.35 ± 0.75
Complex **2**	23.12 ± 0.37/22.97 ± 0.17	87.89 ± 0.84	85.39 ± 0.52
Complex **3**	18.94 ± 0.73/18.98 ± 0.74	94.31 ± 0.78	87.72 ± 0.36
BHT	31.30 ± 0.10/60.00 ± 0.38	not tested	not tested
NDGA	81.02 ± 0.18/82.60 ± 0.17	not tested	not tested
Trolox	not tested	82.80 ± 0.13	91.8 ± 0.17

Each experiment was performed at least in triplicate SD < ±10%.

**Table 7:** The albumin-quenching constants (K_q_) and albumin-binding constants (K) for complexes **1**–**3**.

Compound	ΔI/Io (%)	K_q_ (M^−1^s^−1^)	Κ (M^−1^)
**BSA**			
Complex **1**	75.6	2.28 (±0.10) × 10^13^	3.09 (±0.96) × 10^5^
Complex **2**	92.5	8.70 (±0.43) × 10^13^	6.09 (±0.31) × 10^5^
Complex **3**	97.6	8.45 (±0.31) × 10^13^	5.44 (±0.05) × 10^5^
**HSA**			
Complex **1**	81.7	2.29 (±0.75) × 10^13^	2.86 (±0.11) × 10^5^
Complex **2**	99.4	6.09 (±0.30) × 10^13^	3.87 (±0.42) × 10^5^
Complex **3**	96.5	5.22 (±0.35) × 10^13^	2.42 (±0.17) × 10^5^

**Table 8:** UV-vis spectral features of the interaction of complexes **1**–**3** with CT DNA. UV-band (λ_max_, in nm), percentage of the observed hyper-/hypo-chromism (ΔA/A_0_, in %), blue/red shift of the λ_max_ (Δλ, in nm), and DNA-binding constants (K_b_).

Complex	λ_max_ (nm) (ΔA/A_0_ (%) ^a^, Δλ (nm) ^b^)	K_b_ (M^−1^)
Complex **1**	297 (−7, +1)	2.32 (±0.07) × 10^3^
Complex **2**	285 (+13, +4)	1.77 (±0.67) × 10^4^
Complex **3**	285 (+36, +3)	3.36 (±0.30) × 10^4^

^a^ “+” denotes hyperchromism, “−” denotes hypochromism. ^b^ “+” denotes red shift, “−” denotes blue shift.

**Table 9:** Fluorescence features of the EB-displacement studies: percentage of EB–DNA fluorescence quenching (ΔI/Io, %), Stern–Volmer constants (K_SV_, in M^−1^), and quenching constants of the EB–DNA fluorescence (K_q_, in M^−1^s^−1^) for complexes **1**–**3**.

Complex	ΔI/Io (%)	K_SV_ (M^−1^)	K_q_ (M^−1^s^−1^)
Complex **1**	66.8	6.37 (±0.40) × 10^5^	2.77 (±0.10) × 10^13^
Complex **2**	59.2	6.15 (±0.29) × 10^5^	2.68 (±0.56) × 10^13^
Complex **3**	74.3	1.53 (±0.51) × 10^6^	6.66 (±0.23) × 10^13^

## Data Availability

Not applicable.

## Supplementary Materials

The following supporting information can be downloaded at: https://www.mdpi.com/article/10.3390/ijms24076391/s1.

## Abbreviations

ABTS = 2,2′-azino-bis(3-ethylbenzothiazoline-6-sulfonic acid); Ach = acetylcholine; AChE = acetylcholinesterase; AD = Alzheimer’s disease; ATCI = acetylthiocholine iodide; BHT = butylated hydroxytoluene; BSA = bovine serum albumin; BTCI = S-butyrylthiocholine iodide; BuChE = butyrylcholinesterase; CT = calf-thymus; difl^2−^ = doubly deprotonated diflunisal; DPPH = 1,1-diphenyl-picrylhydrazyl; DTNB = 5,5-dithio-bis-(2-nitrobenzoic acid); EB = ethidium bromide, 3,8-diamino-5-ethyl-6-phenyl-phenanthridinium bromide; eeAChE = electric eel acetylcholinesterase; eqBuChE = equine serum butyrylcholinesterase; FDA = Food and Drug Administration, USA; Hdicl = diclofenac acid; H_2_difl = diflunisal; HSA = human serum albumin; K = SA-binding constant; K_b_ = DNA-binding constant; K_q_ = quenching constant; K_SV_ = Stern-Volmer constant; Nadicl = Sodium diclofenac; NDGA = nordihydroguaiaretic acid; Neo = Neostigmine methyl sulfate; NMDA = N-methyl-D-aspartate; NSAID = non-steroidal anti-inflammatory drug; phen = 1,10-phenanthroline; py = pyridine; *r* = [compound]/[DNA] or [SA] ratio; ROS = reactive oxygen species; SA = serum albumin; SI = selectivity index; SP = senile plaques; trolox = 6-hydroxy-2,5,7,8-tetramethylchromane-2-carboxylic acid; Δ*ν*(COO) = *ν*_asym_(COO) − *ν*_sym_(COO).
