# Supplementary material for: Iron(III) Complexes with Non-Steroidal Anti-Inflammatory Drugs: Structure, Antioxidant and Anticholinergic Activity, and Interaction with Biomolecules
Source: Int J Mol Sci. 2023 Mar 28;24(7):6391. doi: 10.3390/ijms24076391 (PMC10094617; doi:10.3390/ijms24076391)

checkCIF/PLATON report

Structure factors have been supplied for datablock(s) I

THIS REPORT IS FOR GUIDANCE ONLY. IF USED AS PART OF A REVIEW PROCEDURE FOR PUBLICATION, IT SHOULD NOT REPLACE THE EXPERTISE OF AN EXPERIENCED CRYSTALLOGRAPHIC REFEREE.

No syntax errors found.   CIF dictionary   Interpreting this report

Datablock: I

```
Bond precision:  C-C = 0.0050 A          Wavelength=0.71073

Cell:          a=7.0270(16)       b=11.719(3)        c=15.665(4)
               alpha=77.863(13)   beta=88.928(13)    gamma=72.872(12)
Temperature:   295 K

                 Calculated                  Reported
Volume           1204.0(5)                   1204.0(5)
Space group      P -1                        P -1
Hall group       -P 1                        ?
Moiety formula   C52 H34 F4 Fe2 N4 O8, H2 O  C52 H36 F4 Fe2 N4 O9
Sum formula      C52 H36 F4 Fe2 N4 O9        C52 H36 F4 Fe2 N4 O9
Mr               1048.55                     1048.56
Dx,g cm-3        1.446                       1.446
Z                1                           1
Mu (mm-1)        0.679                       0.679
F000             536.0                       536.0
F000'            536.96
h,k,lmax         8,14,19                     8,14,19
Nref             4712                        4635
Tmin,Tmax        0.892,0.928                 0.910,0.930
Tmin'            0.805

Correction method= # Reported T Limits: Tmin=0.910 Tmax=0.930
AbsCorr = NUMERICAL

Data completeness= 0.984          Theta(max)= 25.945

R(reflections)= 0.0478( 3951)     wR2(reflections)= 0.0792( 3951)

S = 1.000                  Npar= 370
```

The following ALERTS were generated. Each ALERT has the format
test-name_ALERT_alert-type_alert-level.
Click on the hyperlinks for more details of the test.

Alert level C

```
PLAT911_ALERT_3_C Missing FCF Refl Between Thmin & STh/L=    0.600         39 Report
```

Alert level G

```
PLAT007_ALERT_5_G Number of Unrefined Donor-H Atoms ..............          2 Report
PLAT042_ALERT_1_G Calc. and Reported MoietyFormula Strings  Differ     Please Check
PLAT300_ALERT_4_G Atom Site Occupancy of F1         Constrained at        0.5 Check
PLAT300_ALERT_4_G Atom Site Occupancy of F3         Constrained at        0.5 Check
PLAT300_ALERT_4_G Atom Site Occupancy of C9         Constrained at        0.5 Check
PLAT300_ALERT_4_G Atom Site Occupancy of C10        Constrained at        0.5 Check
PLAT300_ALERT_4_G Atom Site Occupancy of C12        Constrained at        0.5 Check
PLAT300_ALERT_4_G Atom Site Occupancy of C13        Constrained at        0.5 Check
PLAT300_ALERT_4_G Atom Site Occupancy of C27        Constrained at        0.5 Check
PLAT300_ALERT_4_G Atom Site Occupancy of C28        Constrained at        0.5 Check
PLAT300_ALERT_4_G Atom Site Occupancy of C29        Constrained at        0.5 Check
PLAT300_ALERT_4_G Atom Site Occupancy of C30        Constrained at        0.5 Check
PLAT300_ALERT_4_G Atom Site Occupancy of H101       Constrained at        0.5 Check
PLAT300_ALERT_4_G Atom Site Occupancy of H121       Constrained at        0.5 Check
PLAT300_ALERT_4_G Atom Site Occupancy of H131       Constrained at        0.5 Check
PLAT300_ALERT_4_G Atom Site Occupancy of H281       Constrained at        0.5 Check
PLAT300_ALERT_4_G Atom Site Occupancy of H291       Constrained at        0.5 Check
PLAT300_ALERT_4_G Atom Site Occupancy of H301       Constrained at        0.5 Check
PLAT300_ALERT_4_G Atom Site Occupancy of O5         Constrained at        0.5 Check
PLAT300_ALERT_4_G Atom Site Occupancy of H331       Constrained at        0.5 Check
PLAT300_ALERT_4_G Atom Site Occupancy of H332       Constrained at        0.5 Check
PLAT301_ALERT_3_G Main Residue  Disorder ..............(Resd  1  )        14% Note
PLAT302_ALERT_4_G Anion/Solvent/Minor-Residue Disorder (Resd  2  )       100% Note
PLAT304_ALERT_4_G Non-Integer Number of Atoms in ..... (Resd  2  )       1.50 Check
PLAT769_ALERT_4_G CIF Embedded explicitly supplied scattering data     Please Note
PLAT794_ALERT_5_G Tentative Bond Valency for Fe1       (III)     .       3.08 Info
PLAT808_ALERT_5_G No Parseable SHELXL Style Weighting Scheme Found     Please Check
PLAT882_ALERT_1_G No Datum for _diffrn_reflns_av_unetI/netI ......     Please Do !
PLAT912_ALERT_4_G Missing # of FCF Reflections Above STh/L=  0.600         79 Note
PLAT929_ALERT_5_G No Weight Pars,Obs and Calc R1,wR2,S not Checked          ! Info
PLAT960_ALERT_3_G Number of Intensities with I  <   - 2*sig(I) ...        129 Check
```

   0 **ALERT level A** = Most likely a serious problem - resolve or explain
   0 **ALERT level B** = A potentially serious problem, consider carefully
   1 **ALERT level C** = Check. Ensure it is not caused by an omission or oversight
  31 **ALERT level G** = General information/check it is not something unexpected

   2 ALERT type 1 CIF construction/syntax error, inconsistent or missing data
   0 ALERT type 2 Indicator that the structure model may be wrong or deficient
   3 ALERT type 3 Indicator that the structure quality may be low
  23 ALERT type 4 Improvement, methodology, query or suggestion
   4 ALERT type 5 Informative message, check

It is advisable to attempt to resolve as many as possible of the alerts in all categories. Often the minor alerts point to easily fixed oversights, errors and omissions in your CIF or refinement strategy, so attention to these fine details can be worthwhile. In order to resolve some of the more serious problems it may be necessary to carry out additional measurements or structure refinements. However, the purpose of your study may justify the reported deviations and the more serious of these should normally be commented upon in the discussion or experimental section of a paper or in the "special_details" fields of the CIF. checkCIF was carefully designed to identify outliers and unusual parameters, but every test has its limitations and alerts that are not important in a particular case may appear. Conversely, the absence of alerts does not guarantee there are no aspects of the results needing attention. It is up to the individual to critically assess their own results and, if necessary, seek expert advice.

Publication of your CIF in IUCr journals

A basic structural check has been run on your CIF. These basic checks will be run on all CIFs submitted for publication in IUCr journals (*Acta Crystallographica*, *Journal of Applied Crystallography*, *Journal of Synchrotron Radiation*); however, if you intend to submit to *Acta Crystallographica Section C* or *E* or *IUCrData*, you should make sure that full publication checks are run on the final version of your CIF prior to submission.

Publication of your CIF in other journals

Please refer to the *Notes for Authors* of the relevant journal for any special instructions relating to CIF submission.

PLATON version of 05/12/2020; check.def file version of 05/12/2020

Datablock I - ellipsoid plot

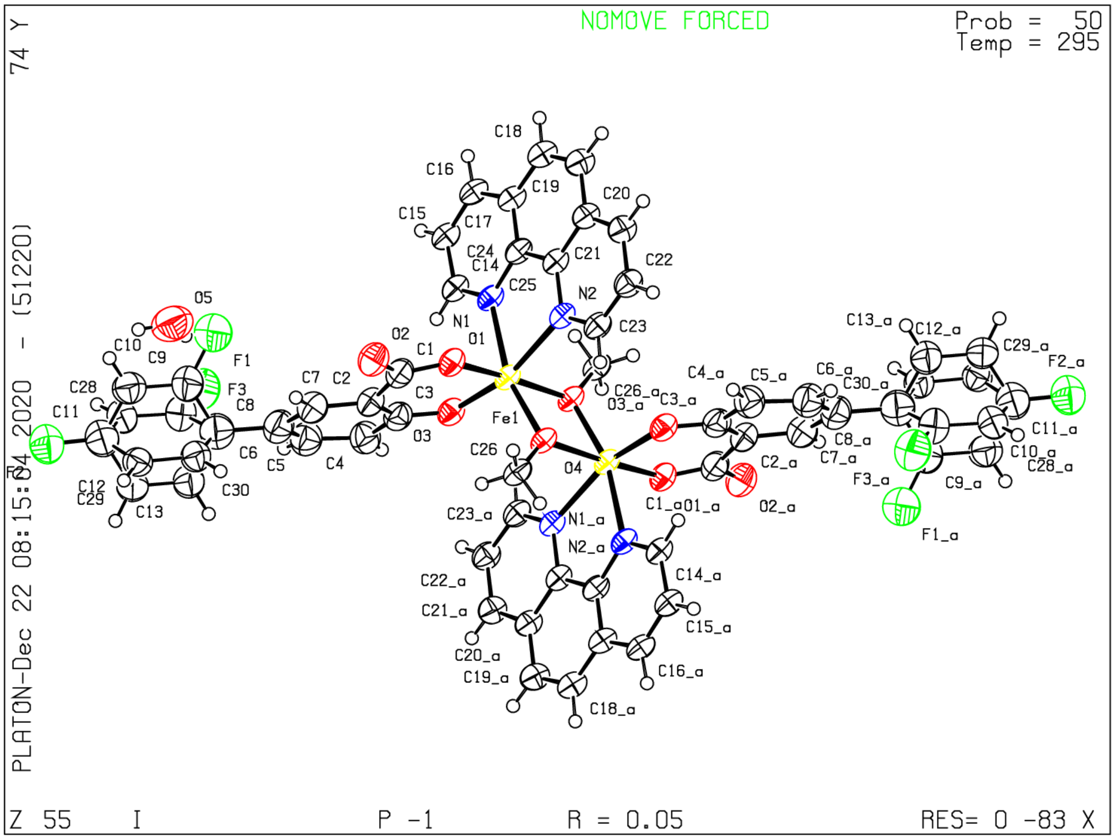

Supplement: Supplementary file 1 [file ijms-24-06391-s001.zip › Checkcif of 1.pdf]
