# Supplementary material for: Iron(III) Complexes with Non-Steroidal Anti-Inflammatory Drugs: Structure, Antioxidant and Anticholinergic Activity, and Interaction with Biomolecules
Source: Int J Mol Sci. 2023 Mar 28;24(7):6391. doi: 10.3390/ijms24076391 (PMC10094617; doi:10.3390/ijms24076391)

checkCIF/PLATON report

Structure factors have been supplied for datablock(s) I

THIS REPORT IS FOR GUIDANCE ONLY. IF USED AS PART OF A REVIEW PROCEDURE FOR PUBLICATION, IT SHOULD NOT REPLACE THE EXPERTISE OF AN EXPERIENCED CRYSTALLOGRAPHIC REFEREE.

No syntax errors found. CIF dictionary Interpreting this report

Datablock: I

```
Bond precision:   C-C = 0.0087 A          Wavelength=0.71073

Cell:            a=18.1059(8)       b=18.6900(8)      c=19.5380(8)
                 alpha=79.916(1)    beta=66.223(2)    gamma=83.277(2)
Temperature:     295 K
```

	Calculated	Reported
Volume	5949.1(4)	5949.1(4)
Space group	P -1	P -1
Hall group	-P 1	?
Moiety formula	C99 H75 Cl12 Fe3 N9 O13, 0.167(C10 H10 N2), 0.667(C5 H5 N), Cl	C104.00 H80.00 Cl13 Fe3 N10.00 O13
Sum formula	C104 H80 Cl13 Fe3 N10 O13	C104.00 H80.00 Cl13 Fe3 N10.00 O13
Mr	2306.18	2306.27
Dx,g cm-3	1.287	1.287
Z	2	2
Mu (mm-1)	0.711	0.711
F000	2354.0	2354.0
F000’	2360.69	
h,k,lmax	21,22,23	21,22,23
Nref	22111	21983
Tmin,Tmax	0.865,0.892	0.890,0.890
Tmin’	0.837	

```
Correction method= # Reported T Limits: Tmin=0.890 Tmax=0.890
AbsCorr = NUMERICAL

Data completeness= 0.994            Theta(max)= 25.476
```

R(reflections)= 0.0483(13266) wR2(reflections)= 0.1309(13266)

S = 1.000 Npar= 1321

The following ALERTS were generated. Each ALERT has the format
test-name_ALERT_alert-type_alert-level.
Click on the hyperlinks for more details of the test.

Alert level C

```
PLAT042_ALERT_1_C Calc. and Reported MoietyFormula Strings  Differ     Please Check
PLAT341_ALERT_3_C Low Bond Precision on  C-C Bonds ...............     0.0087 Ang.
PLAT420_ALERT_2_C D-H Bond Without Acceptor  N4       --H41      .     Please Check
PLAT911_ALERT_3_C Missing FCF Refl Between Thmin & STh/L=    0.600         84 Report
```

Alert level G

```
PLAT002_ALERT_2_G Number of Distance or Angle Restraints on AtSite         18 Note
PLAT003_ALERT_2_G Number of Uiso or Uij Restrained non-H Atoms ...          8 Report
PLAT007_ALERT_5_G Number of Unrefined Donor-H Atoms ..............          6 Report
PLAT300_ALERT_4_G Atom Site Occupancy of N12       Constrained at     0.3333 Check
PLAT300_ALERT_4_G Atom Site Occupancy of C110      Constrained at     0.3333 Check
PLAT300_ALERT_4_G Atom Site Occupancy of C111      Constrained at     0.3333 Check
PLAT300_ALERT_4_G Atom Site Occupancy of C112      Constrained at     0.3333 Check
PLAT300_ALERT_4_G Atom Site Occupancy of C113      Constrained at     0.3333 Check
PLAT300_ALERT_4_G Atom Site Occupancy of C114      Constrained at     0.3333 Check
PLAT300_ALERT_4_G Atom Site Occupancy of H1101     Constrained at     0.3333 Check
PLAT300_ALERT_4_G Atom Site Occupancy of H1111     Constrained at     0.3333 Check
PLAT300_ALERT_4_G Atom Site Occupancy of H1121     Constrained at     0.3333 Check
PLAT300_ALERT_4_G Atom Site Occupancy of H1131     Constrained at     0.3333 Check
PLAT300_ALERT_4_G Atom Site Occupancy of H1141     Constrained at     0.3333 Check
PLAT300_ALERT_4_G Atom Site Occupancy of N10       Constrained at     0.3333 Check
PLAT300_ALERT_4_G Atom Site Occupancy of C100      Constrained at     0.3333 Check
PLAT300_ALERT_4_G Atom Site Occupancy of C101      Constrained at     0.3333 Check
PLAT300_ALERT_4_G Atom Site Occupancy of C102      Constrained at     0.3333 Check
PLAT300_ALERT_4_G Atom Site Occupancy of C103      Constrained at     0.3333 Check
PLAT300_ALERT_4_G Atom Site Occupancy of C104      Constrained at     0.3333 Check
PLAT300_ALERT_4_G Atom Site Occupancy of H1001     Constrained at     0.3333 Check
PLAT300_ALERT_4_G Atom Site Occupancy of H1011     Constrained at     0.3333 Check
PLAT300_ALERT_4_G Atom Site Occupancy of H1021     Constrained at     0.3333 Check
PLAT300_ALERT_4_G Atom Site Occupancy of H1031     Constrained at     0.3333 Check
PLAT300_ALERT_4_G Atom Site Occupancy of H1041     Constrained at     0.3333 Check
PLAT300_ALERT_4_G Atom Site Occupancy of N11       Constrained at     0.3333 Check
PLAT300_ALERT_4_G Atom Site Occupancy of C105      Constrained at     0.3333 Check
PLAT300_ALERT_4_G Atom Site Occupancy of C106      Constrained at     0.3333 Check
PLAT300_ALERT_4_G Atom Site Occupancy of C107      Constrained at     0.3333 Check
PLAT300_ALERT_4_G Atom Site Occupancy of C108      Constrained at     0.3333 Check
PLAT300_ALERT_4_G Atom Site Occupancy of C109      Constrained at     0.3333 Check
PLAT300_ALERT_4_G Atom Site Occupancy of H1051     Constrained at     0.3333 Check
PLAT300_ALERT_4_G Atom Site Occupancy of H1061     Constrained at     0.3333 Check
PLAT300_ALERT_4_G Atom Site Occupancy of H1071     Constrained at     0.3333 Check
PLAT300_ALERT_4_G Atom Site Occupancy of H1081     Constrained at     0.3333 Check
PLAT300_ALERT_4_G Atom Site Occupancy of H1091     Constrained at     0.3333 Check
PLAT300_ALERT_4_G Atom Site Occupancy of Cl13      Constrained at       0.25 Check
PLAT300_ALERT_4_G Atom Site Occupancy of Cl14      Constrained at       0.25 Check
```

```
PLAT300_ALERT_4_G Atom Site Occupancy of Cl15       Constrained at      0.125 Check
PLAT300_ALERT_4_G Atom Site Occupancy of Cl16       Constrained at      0.125 Check
PLAT300_ALERT_4_G Atom Site Occupancy of Cl17       Constrained at      0.125 Check
PLAT300_ALERT_4_G Atom Site Occupancy of Cl18       Constrained at      0.125 Check
PLAT302_ALERT_4_G Anion/Solvent/Minor-Residue Disorder (Resd  2  )       100% Note
PLAT302_ALERT_4_G Anion/Solvent/Minor-Residue Disorder (Resd  3  )       100% Note
PLAT302_ALERT_4_G Anion/Solvent/Minor-Residue Disorder (Resd  4  )       100% Note
PLAT302_ALERT_4_G Anion/Solvent/Minor-Residue Disorder (Resd  5  )       100% Note
PLAT302_ALERT_4_G Anion/Solvent/Minor-Residue Disorder (Resd  6  )       100% Note
PLAT302_ALERT_4_G Anion/Solvent/Minor-Residue Disorder (Resd  7  )       100% Note
PLAT302_ALERT_4_G Anion/Solvent/Minor-Residue Disorder (Resd  8  )       100% Note
PLAT302_ALERT_4_G Anion/Solvent/Minor-Residue Disorder (Resd  9  )       100% Note
PLAT302_ALERT_4_G Anion/Solvent/Minor-Residue Disorder (Resd 10  )       100% Note
PLAT304_ALERT_4_G Non-Integer Number of Atoms in ..... (Resd  2  )       7.33 Check
PLAT304_ALERT_4_G Non-Integer Number of Atoms in ..... (Resd  3  )       3.67 Check
PLAT304_ALERT_4_G Non-Integer Number of Atoms in ..... (Resd  4  )       3.67 Check
PLAT304_ALERT_4_G Non-Integer Number of Atoms in ..... (Resd  5  )       0.25 Check
PLAT304_ALERT_4_G Non-Integer Number of Atoms in ..... (Resd  6  )       0.25 Check
PLAT304_ALERT_4_G Non-Integer Number of Atoms in ..... (Resd  7  )       0.12 Check
PLAT304_ALERT_4_G Non-Integer Number of Atoms in ..... (Resd  8  )       0.12 Check
PLAT304_ALERT_4_G Non-Integer Number of Atoms in ..... (Resd  9  )       0.12 Check
PLAT304_ALERT_4_G Non-Integer Number of Atoms in ..... (Resd 10  )       0.12 Check
PLAT434_ALERT_2_G Short Inter HL..HL Contact Cl10     ..Cl10     .       3.38 Ang.
                                                2-x,-y,1-z  =      2_756 Check
PLAT720_ALERT_4_G Number of Unusual/Non-Standard Labels ..........         19 Note
PLAT769_ALERT_4_G CIF Embedded explicitly supplied scattering data     Please Note
PLAT794_ALERT_5_G Tentative Bond Valency for Fe1       (III)     .       3.02 Info
PLAT794_ALERT_5_G Tentative Bond Valency for Fe2       (III)     .       3.05 Info
PLAT794_ALERT_5_G Tentative Bond Valency for Fe3       (III)     .       3.00 Info
PLAT808_ALERT_5_G No Parseable SHELXL Style Weighting Scheme Found     Please Check
PLAT860_ALERT_3_G Number of Least-Squares Restraints ............         48 Note
PLAT882_ALERT_1_G No Datum for _diffrn_reflns_av_unetI/netI ......     Please Do !
PLAT910_ALERT_3_G Missing # of FCF Reflection(s) Below Theta(Min).          1 Note
PLAT912_ALERT_4_G Missing # of FCF Reflections Above STh/L=  0.600        113 Note
PLAT929_ALERT_5_G No Weight Pars,Obs and Calc R1,wR2,S not Checked          ! Info
PLAT960_ALERT_3_G Number of Intensities with I  <   - 2*sig(I) ...          1 Check
```

---

   0 **ALERT level A** = Most likely a serious problem - resolve or explain
   0 **ALERT level B** = A potentially serious problem, consider carefully
   4 **ALERT level C** = Check. Ensure it is not caused by an omission or oversight
  73 **ALERT level G** = General information/check it is not something unexpected

   2 ALERT type 1 CIF construction/syntax error, inconsistent or missing data
   4 ALERT type 2 Indicator that the structure model may be wrong or deficient
   5 ALERT type 3 Indicator that the structure quality may be low
  60 ALERT type 4 Improvement, methodology, query or suggestion
   6 ALERT type 5 Informative message, check

It is advisable to attempt to resolve as many as possible of the alerts in all categories. Often the minor alerts point to easily fixed oversights, errors and omissions in your CIF or refinement strategy, so attention to these fine details can be worthwhile. In order to resolve some of the more serious problems it may be necessary to carry out additional measurements or structure refinements. However, the purpose of your study may justify the reported deviations and the more serious of these should normally be commented upon in the discussion or experimental section of a paper or in the "special_details" fields of the CIF. checkCIF was carefully designed to identify outliers and unusual parameters, but every test has its limitations and alerts that are not important in a particular case may appear. Conversely, the absence of alerts does not guarantee there are no aspects of the results needing attention. It is up to the individual to critically assess their own results and, if necessary, seek expert advice.

Publication of your CIF in IUCr journals

A basic structural check has been run on your CIF. These basic checks will be run on all CIFs submitted for publication in IUCr journals (*Acta Crystallographica*, *Journal of Applied Crystallography*, *Journal of Synchrotron Radiation*); however, if you intend to submit to *Acta Crystallographica Section C* or *E* or *IUCrData*, you should make sure that full publication checks are run on the final version of your CIF prior to submission.

Publication of your CIF in other journals

Please refer to the *Notes for Authors* of the relevant journal for any special instructions relating to CIF submission.

PLATON version of 28/11/2022; check.def file version of 28/11/2022

Datablock I - ellipsoid plot

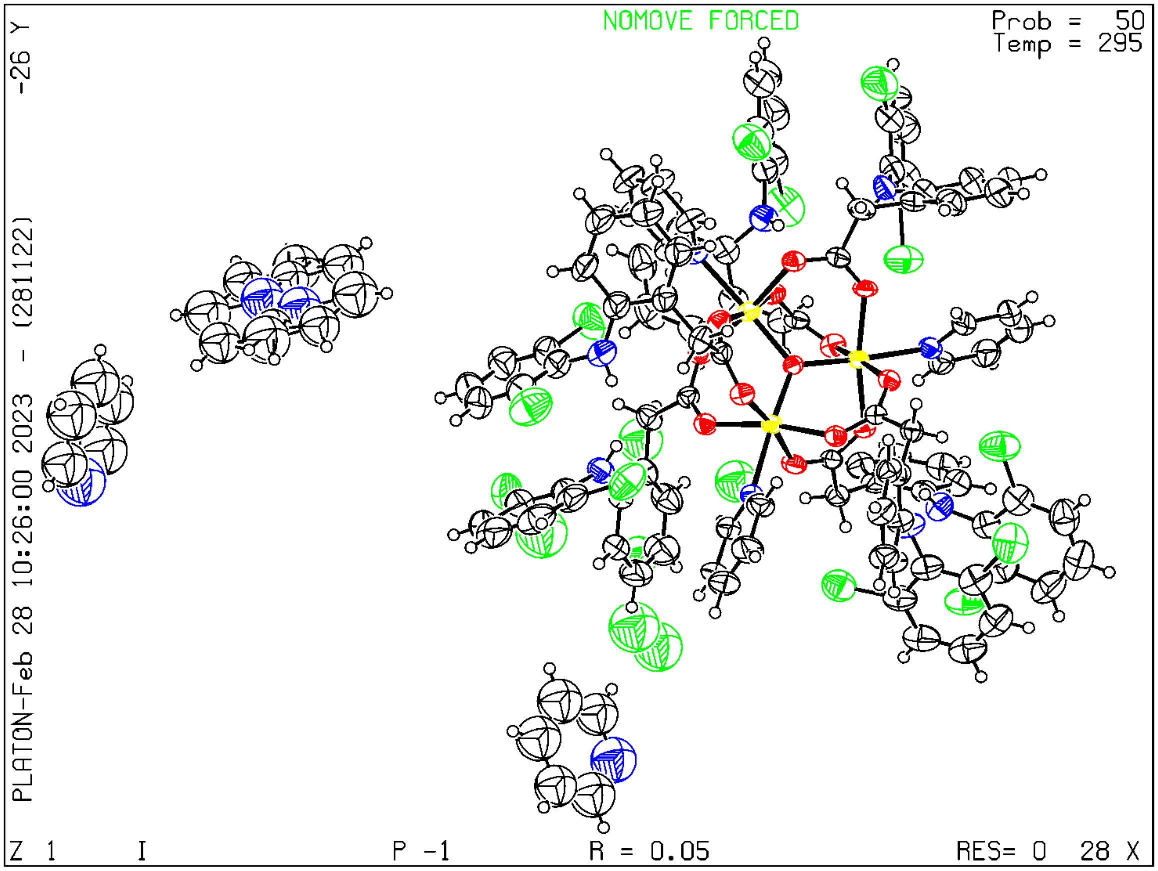

Supplement: Supplementary file 1 [file ijms-24-06391-s001.zip › Checkcif of 2.pdf]
