# Supplementary material for: Iron(III) Complexes with Non-Steroidal Anti-Inflammatory Drugs: Structure, Antioxidant and Anticholinergic Activity, and Interaction with Biomolecules
Source: Int J Mol Sci. 2023 Mar 28;24(7):6391. doi: 10.3390/ijms24076391 (PMC10094617; doi:10.3390/ijms24076391)

checkCIF/PLATON report

Structure factors have been supplied for datablock(s) I

THIS REPORT IS FOR GUIDANCE ONLY. IF USED AS PART OF A REVIEW PROCEDURE FOR PUBLICATION, IT SHOULD NOT REPLACE THE EXPERTISE OF AN EXPERIENCED CRYSTALLOGRAPHIC REFEREE.

No syntax errors found. CIF dictionary Interpreting this report

Datablock: I

Bond precision: C-C = 0.0058 A Wavelength=0.71073

Cell:	a=15.8519(11)	b=18.1237(11)	c=22.7624(16)
	alpha=83.530(3)	beta=84.653(4)	gamma=86.714(4)
Temperature:	295 K		

	Calculated	Reported
Volume	6462.4(8)	6462.4(8)
Space group	P -1	P -1
Hall group	-P 1	?
Moiety formula	2(C87 H72 Cl12 Fe3 N6 O16), 2(C14 H11 Cl2 N O2), 2(Cl4 Fe), 3(C	C102.50 H91 Cl18 Fe4 N7 O20.50
Sum formula	C205 H182 Cl36 Fe8 N14 O41	C102.50 H91 Cl18 Fe4 N7 O20.50
Mr	5220.66	2610.43
Dx,g cm-3	1.342	1.341
Z	1	2
Mu (mm-1)	0.873	0.873
F000	2658.0	2658.0
F000’	2667.01	
h,k,lmax	19,22,28	19,21,28
Nref	25470	24332
Tmin,Tmax	0.846,0.885	0.870,0.880
Tmin’	0.818	

Correction method= # Reported T Limits: Tmin=0.870 Tmax=0.880
AbsCorr = NUMERICAL

Data completeness= 0.955 Theta(max)= 26.021

R(reflections)= 0.0519(16440) wR2(reflections)= 0.0850(16440)

S = 1.000 Npar= 1543

The following ALERTS were generated. Each ALERT has the format
test-name_ALERT_alert-type_alert-level.
Click on the hyperlinks for more details of the test.

Alert level C

```
PLAT029_ALERT_3_C _diffrn_measured_fraction_theta_full value Low .      0.970 Why?
PLAT042_ALERT_1_C Calc. and Reported MoietyFormula Strings  Differ     Please Check
PLAT250_ALERT_2_C Large U3/U1 Ratio for Average U(i,j) Tensor ....        2.4 Note
PLAT420_ALERT_2_C D-H Bond Without Acceptor  N1       --H11      .     Please Check
PLAT420_ALERT_2_C D-H Bond Without Acceptor  N4       --H1185    .     Please Check
PLAT420_ALERT_2_C D-H Bond Without Acceptor  N5       --H1186    .     Please Check
PLAT420_ALERT_2_C D-H Bond Without Acceptor  N6       --H1187    .     Please Check
PLAT420_ALERT_2_C D-H Bond Without Acceptor  N2       --H1188    .     Please Check
PLAT420_ALERT_2_C D-H Bond Without Acceptor  N3       --H31      .     Please Check
PLAT911_ALERT_3_C Missing FCF Refl Between Thmin & STh/L=    0.600        489 Report
```

Alert level G

```
PLAT007_ALERT_5_G Number of Unrefined Donor-H Atoms .............          19 Report
PLAT045_ALERT_1_G Calculated and Reported Z Differ by a Factor ...      0.500 Check
PLAT300_ALERT_4_G Atom Site Occupancy of Cl17       Constrained at        0.5 Check
PLAT300_ALERT_4_G Atom Site Occupancy of Cl18       Constrained at        0.5 Check
PLAT300_ALERT_4_G Atom Site Occupancy of O19        Constrained at        0.5 Check
PLAT300_ALERT_4_G Atom Site Occupancy of O20        Constrained at        0.5 Check
PLAT300_ALERT_4_G Atom Site Occupancy of N7         Constrained at        0.5 Check
PLAT300_ALERT_4_G Atom Site Occupancy of C88        Constrained at        0.5 Check
PLAT300_ALERT_4_G Atom Site Occupancy of C89        Constrained at        0.5 Check
PLAT300_ALERT_4_G Atom Site Occupancy of C90        Constrained at        0.5 Check
PLAT300_ALERT_4_G Atom Site Occupancy of C91        Constrained at        0.5 Check
PLAT300_ALERT_4_G Atom Site Occupancy of C92        Constrained at        0.5 Check
PLAT300_ALERT_4_G Atom Site Occupancy of C93        Constrained at        0.5 Check
PLAT300_ALERT_4_G Atom Site Occupancy of C94        Constrained at        0.5 Check
PLAT300_ALERT_4_G Atom Site Occupancy of C95        Constrained at        0.5 Check
PLAT300_ALERT_4_G Atom Site Occupancy of C96        Constrained at        0.5 Check
PLAT300_ALERT_4_G Atom Site Occupancy of C97        Constrained at        0.5 Check
PLAT300_ALERT_4_G Atom Site Occupancy of C98        Constrained at        0.5 Check
PLAT300_ALERT_4_G Atom Site Occupancy of C99        Constrained at        0.5 Check
PLAT300_ALERT_4_G Atom Site Occupancy of C100       Constrained at        0.5 Check
PLAT300_ALERT_4_G Atom Site Occupancy of C101       Constrained at        0.5 Check
PLAT300_ALERT_4_G Atom Site Occupancy of H1001      Constrained at        0.5 Check
PLAT300_ALERT_4_G Atom Site Occupancy of H1189      Constrained at        0.5 Check
PLAT300_ALERT_4_G Atom Site Occupancy of H71        Constrained at        0.5 Check
PLAT300_ALERT_4_G Atom Site Occupancy of H891       Constrained at        0.5 Check
PLAT300_ALERT_4_G Atom Site Occupancy of H892       Constrained at        0.5 Check
PLAT300_ALERT_4_G Atom Site Occupancy of H911       Constrained at        0.5 Check
PLAT300_ALERT_4_G Atom Site Occupancy of H921       Constrained at        0.5 Check
PLAT300_ALERT_4_G Atom Site Occupancy of H931       Constrained at        0.5 Check
PLAT300_ALERT_4_G Atom Site Occupancy of H941       Constrained at        0.5 Check
PLAT300_ALERT_4_G Atom Site Occupancy of H981       Constrained at        0.5 Check
PLAT300_ALERT_4_G Atom Site Occupancy of H991       Constrained at        0.5 Check
```

```
PLAT300_ALERT_4_G Atom Site Occupancy of Cl19       Constrained at        0.5 Check
PLAT300_ALERT_4_G Atom Site Occupancy of Cl20       Constrained at        0.5 Check
PLAT300_ALERT_4_G Atom Site Occupancy of O21        Constrained at        0.5 Check
PLAT300_ALERT_4_G Atom Site Occupancy of O22        Constrained at        0.5 Check
PLAT300_ALERT_4_G Atom Site Occupancy of N8         Constrained at        0.5 Check
PLAT300_ALERT_4_G Atom Site Occupancy of C102       Constrained at        0.5 Check
PLAT300_ALERT_4_G Atom Site Occupancy of C103       Constrained at        0.5 Check
PLAT300_ALERT_4_G Atom Site Occupancy of C104       Constrained at        0.5 Check
PLAT300_ALERT_4_G Atom Site Occupancy of C105       Constrained at        0.5 Check
PLAT300_ALERT_4_G Atom Site Occupancy of C106       Constrained at        0.5 Check
PLAT300_ALERT_4_G Atom Site Occupancy of C107       Constrained at        0.5 Check
PLAT300_ALERT_4_G Atom Site Occupancy of C108       Constrained at        0.5 Check
PLAT300_ALERT_4_G Atom Site Occupancy of C109       Constrained at        0.5 Check
PLAT300_ALERT_4_G Atom Site Occupancy of C110       Constrained at        0.5 Check
PLAT300_ALERT_4_G Atom Site Occupancy of C111       Constrained at        0.5 Check
PLAT300_ALERT_4_G Atom Site Occupancy of C112       Constrained at        0.5 Check
PLAT300_ALERT_4_G Atom Site Occupancy of C113       Constrained at        0.5 Check
PLAT300_ALERT_4_G Atom Site Occupancy of C114       Constrained at        0.5 Check
PLAT300_ALERT_4_G Atom Site Occupancy of C115       Constrained at        0.5 Check
PLAT300_ALERT_4_G Atom Site Occupancy of H1031      Constrained at        0.5 Check
PLAT300_ALERT_4_G Atom Site Occupancy of H1032      Constrained at        0.5 Check
PLAT300_ALERT_4_G Atom Site Occupancy of H1051      Constrained at        0.5 Check
PLAT300_ALERT_4_G Atom Site Occupancy of H1061      Constrained at        0.5 Check
PLAT300_ALERT_4_G Atom Site Occupancy of H1071      Constrained at        0.5 Check
PLAT300_ALERT_4_G Atom Site Occupancy of H1081      Constrained at        0.5 Check
PLAT300_ALERT_4_G Atom Site Occupancy of H1121      Constrained at        0.5 Check
PLAT300_ALERT_4_G Atom Site Occupancy of H1131      Constrained at        0.5 Check
PLAT300_ALERT_4_G Atom Site Occupancy of H1141      Constrained at        0.5 Check
PLAT300_ALERT_4_G Atom Site Occupancy of H1184      Constrained at        0.5 Check
PLAT300_ALERT_4_G Atom Site Occupancy of H221       Constrained at        0.5 Check
PLAT300_ALERT_4_G Atom Site Occupancy of O18        Constrained at        0.5 Check
PLAT300_ALERT_4_G Atom Site Occupancy of C117       Constrained at        0.5 Check
PLAT300_ALERT_4_G Atom Site Occupancy of H1171      Constrained at        0.5 Check
PLAT300_ALERT_4_G Atom Site Occupancy of H1172      Constrained at        0.5 Check
PLAT300_ALERT_4_G Atom Site Occupancy of H1173      Constrained at        0.5 Check
PLAT300_ALERT_4_G Atom Site Occupancy of H1190      Constrained at        0.5 Check
PLAT300_ALERT_4_G Atom Site Occupancy of H232       Constrained at        0.5 Check
PLAT300_ALERT_4_G Atom Site Occupancy of H234       Constrained at        0.5 Check
PLAT300_ALERT_4_G Atom Site Occupancy of O24        Constrained at        0.5 Check
PLAT300_ALERT_4_G Atom Site Occupancy of H243       Constrained at        0.5 Check
PLAT300_ALERT_4_G Atom Site Occupancy of H244       Constrained at        0.5 Check
PLAT302_ALERT_4_G Anion/Solvent/Minor-Residue Disorder (Resd  2  )       100% Note
PLAT302_ALERT_4_G Anion/Solvent/Minor-Residue Disorder (Resd  3  )       100% Note
PLAT302_ALERT_4_G Anion/Solvent/Minor-Residue Disorder (Resd  6  )       100% Note
PLAT302_ALERT_4_G Anion/Solvent/Minor-Residue Disorder (Resd  8  )       100% Note
PLAT304_ALERT_4_G Non-Integer Number of Atoms in ..... (Resd  8  )       1.50 Check
PLAT413_ALERT_2_G Short Inter XH3 .. XHn     H1171    ..H22      .       2.10 Ang.
                                                        x,y,z  =      1_555 Check
PLAT413_ALERT_2_G Short Inter XH3 .. XHn     H1173    ..H752     .       2.09 Ang.
                                                        x,y,z  =      1_555 Check
PLAT417_ALERT_2_G Short Inter D-H..H-D       H91      ..H221     .       2.09 Ang.
                                                        x,y,z  =      1_555 Check
PLAT432_ALERT_2_G Short Inter X...Y Contact  Cl17     ..C10      .       3.19 Ang.
                                                        x,y,z  =      1_555 Check
PLAT432_ALERT_2_G Short Inter X...Y Contact  O21      ..C106     .       2.49 Ang.
                                                    -x,1-y,-z  =      2_565 Check
PLAT432_ALERT_2_G Short Inter X...Y Contact  O21      ..C105     .       2.50 Ang.
```

```
                                                  -x,1-y,-z  =      2_565 Check
PLAT432_ALERT_2_G Short Inter X...Y Contact  O21      ..C104     .       2.74 Ang.
                                                  -x,1-y,-z  =      2_565 Check
PLAT432_ALERT_2_G Short Inter X...Y Contact  O21      ..C107     .       2.76 Ang.
                                                  -x,1-y,-z  =      2_565 Check
PLAT432_ALERT_2_G Short Inter X...Y Contact  O21      ..C109     .       2.97 Ang.
                                                  -x,1-y,-z  =      2_565 Check
PLAT432_ALERT_2_G Short Inter X...Y Contact  O21      ..C108     .       2.99 Ang.
                                                  -x,1-y,-z  =      2_565 Check
PLAT432_ALERT_2_G Short Inter X...Y Contact  O24      ..C114     .       2.97 Ang.
                                                      x,y,z  =      1_555 Check
PLAT432_ALERT_2_G Short Inter X...Y Contact  O24      ..C116     .       2.99 Ang.
                                                    x,1+y,z  =      1_565 Check
PLAT432_ALERT_2_G Short Inter X...Y Contact  C104     ..C117     .       3.18 Ang.
                                                      x,y,z  =      1_555 Check
PLAT432_ALERT_2_G Short Inter X...Y Contact  C105     ..C117     .       2.38 Ang.
                                                      x,y,z  =      1_555 Check
PLAT432_ALERT_2_G Short Inter X...Y Contact  C106     ..C117     .       3.16 Ang.
                                                      x,y,z  =      1_555 Check
PLAT434_ALERT_2_G Short Inter HL..HL Contact Cl5      ..Cl17     .       3.33 Ang.
                                                      x,y,z  =      1_555 Check
PLAT434_ALERT_2_G Short Inter HL..HL Contact Cl6      ..Cl20     .       3.27 Ang.
                                                      x,y,z  =      1_555 Check
PLAT720_ALERT_4_G Number of Unusual/Non-Standard Labels ..........         24 Note
PLAT769_ALERT_4_G CIF Embedded explicitly supplied scattering data     Please Note
PLAT794_ALERT_5_G Tentative Bond Valency for Fe1       (III)     .       3.17 Info
PLAT794_ALERT_5_G Tentative Bond Valency for Fe2       (III)     .       2.91 Info
PLAT794_ALERT_5_G Tentative Bond Valency for Fe3       (III)     .       3.02 Info
PLAT808_ALERT_5_G No Parseable SHELXL Style Weighting Scheme Found     Please Check
PLAT860_ALERT_3_G Number of Least-Squares Restraints ............          3 Note
PLAT882_ALERT_1_G No Datum for _diffrn_reflns_av_unetI/netI ......     Please Do !
PLAT910_ALERT_3_G Missing # of FCF Reflection(s) Below Theta(Min).          3 Note
PLAT912_ALERT_4_G Missing # of FCF Reflections Above STh/L=  0.600        696 Note
PLAT913_ALERT_3_G Missing # of Very Strong Reflections in FCF ....          1 Note
PLAT929_ALERT_5_G No Weight Pars,Obs and Calc R1,wR2,S not Checked          ! Info
PLAT940_ALERT_3_G Fsqd Refinement With I > n * Sigma(I) Only .....     Please Check
PLAT960_ALERT_3_G Number of Intensities with I  <   - 2*sig(I) ...        102 Check
PLAT992_ALERT_5_G Repd & Actual _reflns_number_gt Values Differ by          4 Check
```

```
  0 ALERT level A = Most likely a serious problem - resolve or explain
  0 ALERT level B = A potentially serious problem, consider carefully
 10 ALERT level C = Check. Ensure it is not caused by an omission or oversight
110 ALERT level G = General information/check it is not something unexpected

  3 ALERT type 1 CIF construction/syntax error, inconsistent or missing data
 24 ALERT type 2 Indicator that the structure model may be wrong or deficient
  7 ALERT type 3 Indicator that the structure quality may be low
 79 ALERT type 4 Improvement, methodology, query or suggestion
  7 ALERT type 5 Informative message, check
```

It is advisable to attempt to resolve as many as possible of the alerts in all categories. Often the minor alerts point to easily fixed oversights, errors and omissions in your CIF or refinement strategy, so attention to these fine details can be worthwhile. In order to resolve some of the more serious problems it may be necessary to carry out additional measurements or structure refinements. However, the purpose of your study may justify the reported deviations and the more serious of these should normally be commented upon in the discussion or experimental section of a paper or in the "special_details" fields of the CIF. checkCIF was carefully designed to identify outliers and unusual parameters, but every test has its limitations and alerts that are not important in a particular case may appear. Conversely, the absence of alerts does not guarantee there are no aspects of the results needing attention. It is up to the individual to critically assess their own results and, if necessary, seek expert advice.

Publication of your CIF in IUCr journals

A basic structural check has been run on your CIF. These basic checks will be run on all CIFs submitted for publication in IUCr journals (*Acta Crystallographica*, *Journal of Applied Crystallography*, *Journal of Synchrotron Radiation*); however, if you intend to submit to *Acta Crystallographica Section C* or *E* or *IUCrData*, you should make sure that full publication checks are run on the final version of your CIF prior to submission.

Publication of your CIF in other journals

Please refer to the *Notes for Authors* of the relevant journal for any special instructions relating to CIF submission.

PLATON version of 28/11/2022; check.def file version of 28/11/2022

Datablock I - ellipsoid plot

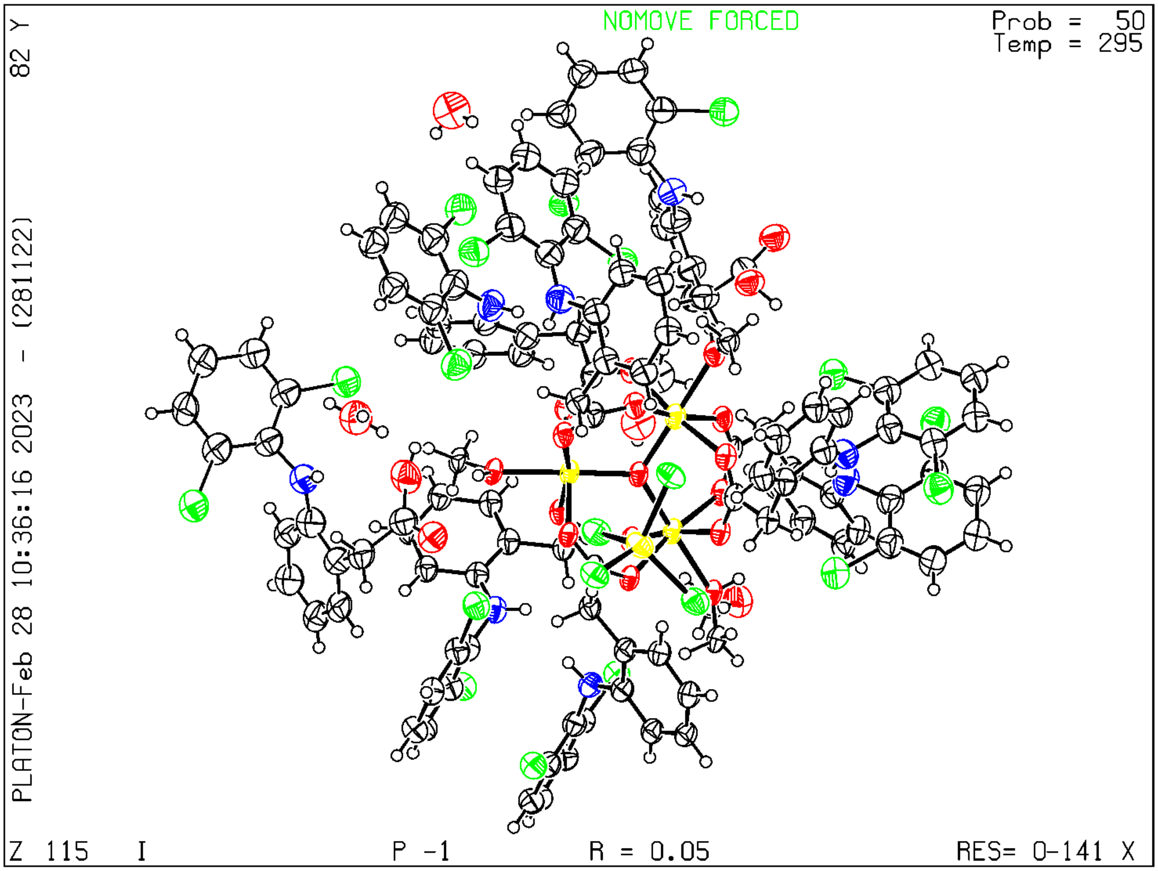

Supplement: Supplementary file 1 [file ijms-24-06391-s001.zip › Checkcif of 3.pdf]
