# Supplementary material for: Iron(III) Complexes with Non-Steroidal Anti-Inflammatory Drugs: Structure, Antioxidant and Anticholinergic Activity, and Interaction with Biomolecules
Source: Int J Mol Sci. 2023 Mar 28;24(7):6391. doi: 10.3390/ijms24076391 (PMC10094617; doi:10.3390/ijms24076391)
Supplement: Supplementary file 1 [file ijms-24-06391-s001.zip › ijms-2299359 - Supplementary.pdf]

Iron(III) Complexes with Non-Steroidal Anti-Inflammatory Drugs: Structure, Antioxidant and Anticholinergic Activity, and Interaction with Biomolecules

Filitsa Dimiza [1], Amalia Barmpa [1], Antonios Chronakis [2], Antonios G. Hatzidimitriou [1], Yiannis Sanakis [3], Athanasios N. Papadopoulos [2] and George Psomas [1,*]

[1] Department of General and Inorganic Chemistry, Faculty of Chemistry, Aristotle University of Thessaloniki, GR-54124 Thessaloniki, Greece

[2] Department of Nutritional Sciences and Dietetics, International Hellenic University, GR-57400 Thessaloniki, Greece

[3] Institute of Nanoscience and Nanotechnology, National Centre of Scientific Research "Demokritos", GR-15310 Agia Paraskevi, Greece

* Correspondence: gepsomas@chem.auth.gr

Supplementary Materials

* Corresponding author's e–mail: gepsomas@chem.auth.gr

INDEX

Content	Page
**Table S1.** Crystal determination data for complexes **1-3**	3
**Table S2**. Selected bond lengths and bond angles for complex **1.**	4
**Table S3**. Selected bond lengths and bond angles for complex **2**.	5
**Table S4**. H-bonding interactions in complexes **2** and **3**.	6
**Table S5**. Selected bond lengths and bond angles for complex **3**.	7
**Figure S1**. IR spectra (KBr pellet) of complexes **1**-**3**.	8
**Figure S2**. Stern-Volmer quenching plots of BSA for complexes **1**-**3**.	9
**Figure S3**. Stern-Volmer quenching plots of HSA for complexes **1**-**3**.	9
**Figure S4**. Scatchard plots of BSA for complexes **1-3**.	10
**Figure S5**. Scatchard plots of HSA for complexes **1**-**3**.	10
**Figure S6**. UV–vis spectra of a DMSO solution of complexes **1** and **2**, in the presence of increasing amounts of CT DNA.	11
**Figure S7**. Plots of [DNA]/(ε_A-ε_f) *versus* [DNA] for complexes **1**-**3.**	11
**Figure S8**. Stern-Volmer quenching plots of EB-DNA fluorescence for complexes **1**-**3**.	12

Table S1. Crystal determination data for complexes $[Fe_2(difl)_4(phen)_2(MeO)_2]\cdot H_2O$, **(1),** $[Fe_3O(dicl)_6(py)_3]Cl\cdot py$, **(2)** and $[Fe_3O(dicl)_6(MeOH)_3][FeCl_4]\cdot Hdicl\cdot 1.5MeOH\cdot H_2O$, **(3).**

	Complex **1**	Complex **2**	Complex **3**
Crystal data			
Chemical Formula sum	$C_{52}H_{36}F_4Fe_2N_4O_9$	$C_{104.00}H_{80.00}Cl_{13}Fe_3N_{10.00}O_{13}$	$C_{102.50}H_{91}Cl_{18}Fe_4N_7O_{20.50}$
Chemical Formula moiety	$C_{52}H_{34}F_4Fe_2N_4O_8$, H_2O	$C_{99}H_{75}Cl_{12}Fe_3N_9O_{13}$, C_5H_5N, Cl	$C_{87}H_{72}Cl_{12}Fe_3N_6O_{16}$, $C_{14}H_{11}Cl_2NO_2$, Cl_4Fe, $1.5(CH_4O)$, H_2O
M_r	1048.56	2287.00	2610.43
Crystal system	Triclinic	Triclinic	Triclinic
Space group	*P-1*	*P-1*	*P-1*
Temperature (K)	295	295	295
a (Å)	7.0270 (16)	18.1059 (8)	15.8519 (11)
b (Å)	11.719 (3)	18.6900 (8)	18.1237 (11),
c (Å)	15.665 (4)	19.5380 (8)	22.7624 (16)
α (°)	77.863 (13)	79.916 (1)	83.530 (3),
β (°)	88.928 (13)	66.223 (2)	84.653 (4),
γ (°)	72.872 (12)	83.277 (2)	86.714 (4)
V (Å^3)	1204.0 (5)	5949.1 (4)	6462.4 (8)
Z	1	2	2
Radiation type	Mo$K\alpha$	Mo$K\alpha$	Mo$K\alpha$
(μ) mm^{-1}	0.68	0.71	0.87
Crystal size, mm	0.32 × 0.14 × 0.11	0.25 × 0.17 × 0.16	0.27 × 0.19 × 0.15
Data collection			
Diffractometer		Bruker Kappa Apex2	
Absorption correction		Numerical / Analytical Absorption (De Meulenaer & Tompa, 1965)	
T_{min}, T_{max}	0.91, 0.93	0.89, 0.89	0.87, 0.88
No. of measured reflections	27894	82916	81474
No. of independent reflections	4635	21983	24332
No. of observed [I > 2.0σ(I)] reflections	3951	13266	16440
R_{int}	0.026	0.037	0.026
(sin θ/λ)$_{max}$ (Å^{-1})	0.616	0.605	0.617
Refinement			
$R[F^2 > 2\sigma(F^2)]$, $wR(F^2)$, S	0.048, 0.076, 1.00	0.048, 0.110, 1.00	0.052, 0.077, 1.00
No. of reflections	3951	13266	16440
No. of parameters	370	1321	1543
No. of restraints	-	48	3
H-atom treatment		H-atom parameters constrained	
$\Delta\rho_{max}$, $\Delta\rho_{min}$ (e Å^{-3})	0.33, −0.32	0.47, −0.45	0.69, −0.45

Table S2. Selected bond lengths (Å) and bond angles (°) for complex **1**.

**Bond**	**Length (Å)**	**Bond**	**Length (Å)**
Fe(1)-O(4^{i})	1.9984(19)	Fe(1)-N(1)	2.200(2)
Fe(1)-O(1)	1.920(2)	Fe(1)-N(2)	2.191(3)
Fe(1)-O(4)	1.9726(19)	Fe(1)-O(3)	1.928(2)
Fe(1)… Fe(1^{i})	3.119		
O(1)—C(1)	1.273 (4)	O(3)—C(3)	1.295 (4)
O(2)—C(1)	1.264 (4)	O(4)—C(26)	1.405 (3)
**Bonds**	**Angle (°)**	**Bonds**	**Angle (°)**
O(4^{i})-Fe(1)-N(1)	94.36 (9)	O(1)-Fe(1)-O(3)	89.66 (9)
O(4^{i})-Fe(1)-N(2)	89.39 (9)	O(4^{i})-Fe(1)-O(4)	76.46 (9)
N(1)-Fe(1)-N(2)	75.03 (9)	N(1)-Fe(1)-O(4)	164.56 (9)
O(4^{i})-Fe(1)-O(1)	172.52 (8)	N(2)-Fe(1)-O(4)	92.27 (9)
N(1)-Fe(1)-O(1)	92.17 (9)	O(1)-Fe(1)-O(4)	96.36 (8)
N(2)-Fe(1)-O(1)	88.77 (9)	O(3)-Fe(1)-O(4)	104.73 (9)
O(4^{i})-Fe(1)-O(3)	94.19 (9)	N(1)-Fe(1)-O(3)	88.11 (9)
N(2)-Fe(1)-O(3)	163.00 (8)		
Fe(1)-O(3)-C(3)	127.4 (2)	Fe(1^{i})-O(4)-Fe(1)	103.54 (9)

Symmetry code : (i) -x+1, -y+1, -z+1.

Table S3. Selected bond lengths (Å) and bond angles (°) for complex **2**.

Bond	Length (Å)	Bond	Length (Å)	Bond	Length (Å)
Fe1—O1	2.019 (2)	Fe2—O2	2.012 (3)	Fe3—O4	2.039 (3)
Fe1—O3	2.018 (3)	Fe2—O6	2.017 (3)	Fe3—O5	2.021 (3)
Fe1—O7	2.028 (3)	Fe2—O8	2.039 (3)	Fe3—O10	2.041 (3)
Fe1—O9	2.029 (2)	Fe2—O11	1.995 (3)	Fe3—O12	2.020 (3)
Fe1—O13	1.910 (2)	Fe2—O13	1.922 (2)	Fe3—O13	1.890 (2)
Fe1—N1	2.157 (3)	Fe2—N2	2.149 (3)	Fe3—N3	2.179 (3)
Fe1…Fe2	3.311	Fe2…Fe3	3.302	Fe1…Fe3	3.296

Bonds	Angle (°)	Bonds	Angle (°)	Bonds	Angle (°)
O1—Fe1—O3	90.83 (11)	O2—Fe2—O6	170.94 (11)	O4—Fe3—O5	170.17 (10)
O1—Fe1—O7	95.00 (12)	O2—Fe2—O8	89.49 (11)	O4—Fe3—O10	92.54 (11)
O3—Fe1—O7	171.83 (11)	O6—Fe2—O8	84.62 (11)	O5—Fe3—O10	85.99 (11)
O1—Fe1—O9	168.85 (11)	O2—Fe2—O11	89.98 (12)	O4—Fe3—O12	90.80 (11)
O3—Fe1—O9	88.68 (11)	O6—Fe2—O11	94.98 (12)	O5—Fe3—O12	89.24 (11)
O7—Fe1—O9	84.50 (11)	O8—Fe2—O11	172.72 (11)	O10—Fe3—O12	170.68 (11)
O1—Fe1—O13	94.51 (11)	O2—Fe2—O13	95.22 (11)	O4—Fe3—O13	93.68 (10)
O3—Fe1—O13	93.93 (11)	O6—Fe2—O13	92.08 (11)	O5—Fe3—O13	96.09 (11)
O7—Fe1—O13	91.33 (11)	O8—Fe2—O13	94.08 (11)	O10—Fe3—O13	92.66 (11)
O9—Fe1—O13	96.64 (10)	O11—Fe2—O13	93.20 (11)	O12—Fe3—O13	95.80 (11)
O1—Fe1—N1	82.49 (12)	O2—Fe2—N2	86.59 (13)	O4—Fe3—N3	83.76 (12)
O3—Fe1—N1	88.36 (13)	O6—Fe2—N2	86.26 (12)	O5—Fe3—N3	86.43 (12)
O7—Fe1—N1	86.73 (13)	O8—Fe2—N2	87.36 (12)	O10—Fe3—N3	84.99 (12)
O9—Fe1—N1	86.36 (11)	O11—Fe2—N2	85.37 (12)	O12—Fe3—N3	86.73 (12)
O13—Fe1—N1	176.26 (12)	O13—Fe2—N2	177.70 (12)	O13—Fe3—N3	176.44 (12)
Fe2—O13—Fe1	119.54 (12)	Fe2—O13—Fe3	120.05 (13)	Fe1—O13—Fe3	120.29 (13)

Table S4. H-bonding interactions in complexes **2** and **3**.

D*—H···*A	***D*—H (Å)**	**H···*A* (Å)**	***D*···*A* (Å)**	***D*—H···*A* (º)**
**2**				
N6—H61···O5	0.84	2.56	3.143 (5)	127
N7—H71···O8	0.84	2.38	3.005 (5)	132
N8—H81···O9	0.85	2.46	3.097 (5)	133
N5—H1135···O4	0.84	2.54	3.123 (5)	127
**3**				
N7—H71···O20	0.87	2.42	2.998 (8)	124
O8—H81···O20	0.93	2.35	3.189 (8)	149
O9—H91···O22	0.85	1.95	2.645 (8)	139
O17—H171···O10	0.73	1.97	2.603 (8)	146
N8—H1184···O21	0.87	2.34	3.076 (8)	142

Table S5. Selected bond lengths (Å) and bond angles (°) for complex **3**.

Bond	Length (Å)	Bond	Length (Å)	Bond	Length (Å)
Fe1—O1	1.863 (2)	Fe2—O1	1.926 (2)	Fe3—O1	1.905 (2)
Fe1—O2	2.019 (2)	Fe2—O3	2.060 (2)	Fe3—O5	2.024 (2)
Fe1—O4	2.013 (2)	Fe2—O7	2.050 (3)	Fe3—O6	2.037 (2)
Fe1—O8	2.057 (2)	Fe2—O9	2.078 (2)	Fe3—O10	2.064 (2)
Fe1—O11	2.002 (2)	Fe2—O12	2.026 (2)	Fe3—O14	2.029 (2)
Fe1—O13	2.051 (2)	Fe2—O16	2.041 (2)	Fe3—O15	2.042 (2)
Fe4—Cl1	2.1748 (11)	Fe4—Cl2	2.1483 (12)		
Fe4—Cl4	2.1857 (12)	Fe4—Cl3	2.1722 (11)		
Fe1…Fe2	3.266	Fe2…Fe3	3.310	Fe1…Fe3	3.287

Bonds	Angle (°)	Bonds	Angle (°)	Bonds	Angle (°)
O1—Fe1—O2	93.31 (10)	O1—Fe2—O3	92.29 (10)	O1—Fe3—O5	94.93 (9)
O1—Fe1—O4	94.99 (9)	O1—Fe2—O7	93.19 (10)	O1—Fe3—O6	93.69 (9)
O2—Fe1—O4	91.69 (9)	O3—Fe2—O7	173.37 (10)	O5—Fe3—O6	171.38 (9)
O1—Fe1—O8	177.10 (9)	O1—Fe2—O9	179.15 (9)	O1—Fe3—O10	176.72 (10)
O2—Fe1—O8	86.04 (9)	O3—Fe2—O9	87.49 (10)	O5—Fe3—O10	88.34 (9)
O4—Fe1—O8	82.21 (8)	O7—Fe2—O9	87.08 (10)	O6—Fe3—O10	83.04 (9)
O1—Fe1—O11	97.53 (9)	O1—Fe2—O12	93.49 (9)	O1—Fe3—O14	92.94 (9)
O2—Fe1—O11	89.47 (10)	O3—Fe2—O12	93.43 (10)	O5—Fe3—O14	92.88 (9)
O4—Fe1—O11	167.34 (9)	O7—Fe2—O12	89.96 (9)	O6—Fe3—O14	86.82 (9)
O8—Fe1—O11	85.29 (9)	O9—Fe2—O12	85.70 (9)	O10—Fe3—O14	86.55 (9)
O1—Fe1—O13	91.03 (9)	O1—Fe2—O16	94.74 (9)	O1—Fe3—O15	94.73 (10)
O2—Fe1—O13	173.90 (9)	O3—Fe2—O16	86.97 (9)	O5—Fe3—O15	86.51 (9)
O4—Fe1—O13	92.20 (9)	O7—Fe2—O16	88.86 (9)	O6—Fe3—O15	92.64 (9)
O8—Fe1—O13	89.84 (9)	O9—Fe2—O16	86.07 (9)	O10—Fe3—O15	85.79 (9)
O11—Fe1—O13	85.71 (9)	O12—Fe2—O16	171.74 (10)	O14—Fe3—O15	172.32 (9)
Cl1—Fe4—Cl2	109.79 (5)	Cl1—Fe4—Cl4	113.78 (5)	Cl2—Fe4—Cl4	108.04 (4)
Cl1—Fe4—Cl3	107.38 (4)	Cl2—Fe4—Cl3	111.61 (5)	Cl3—Fe4—Cl4	106.25 (5)
Fe2—O1—Fe3	119.54(11)	Fe2—O1—Fe1	119.04(11)	Fe3—O1—Fe1	121.40 (12)

1

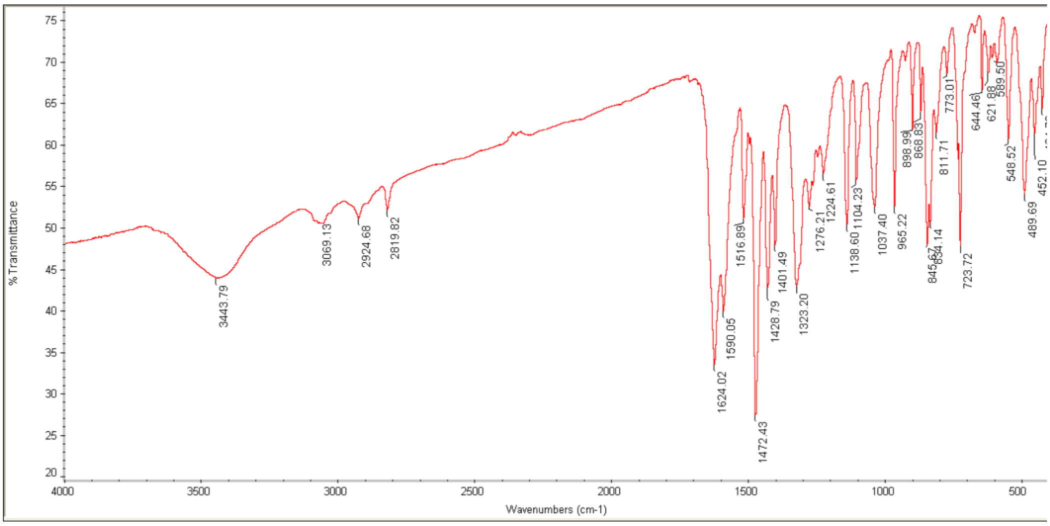

2

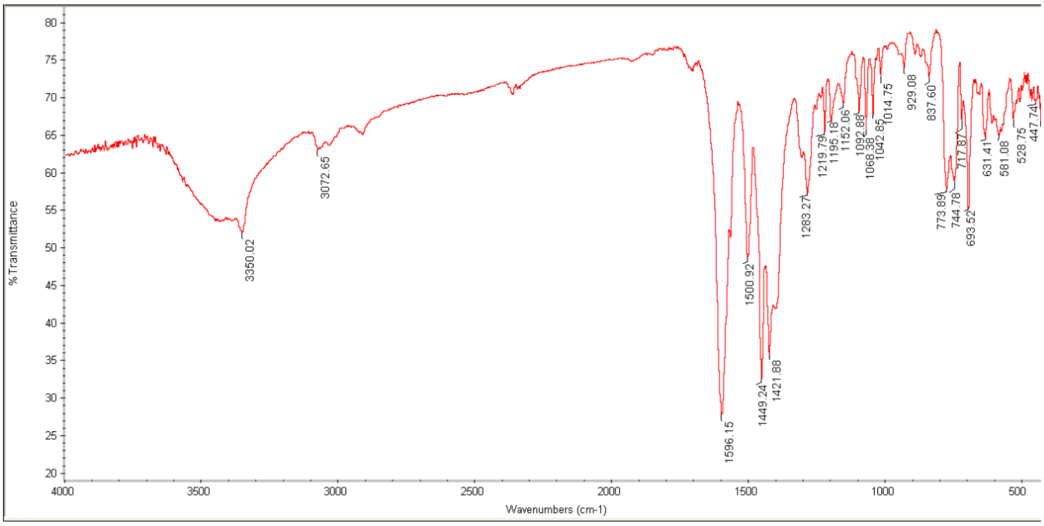

3

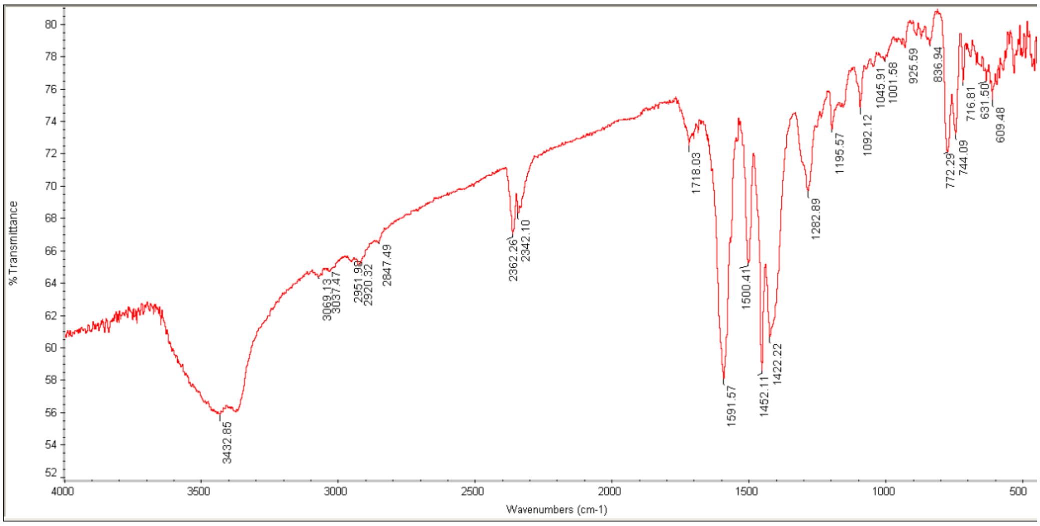

Figure S1. IR spectra (KBr pellet) of complexes **1-3**.

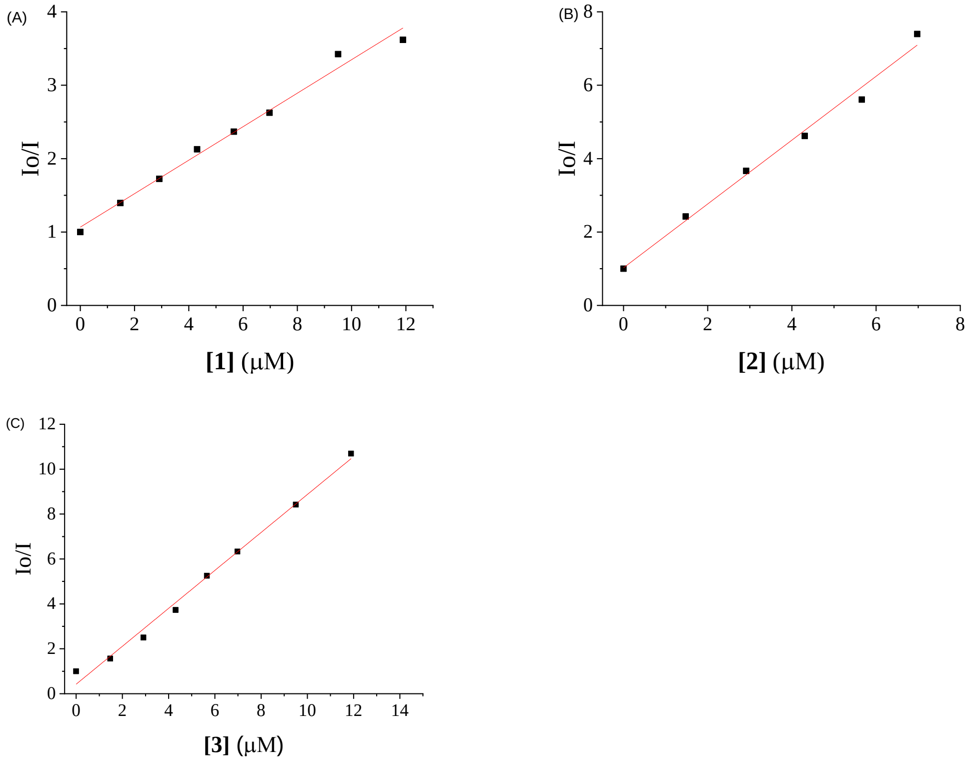

Figure S2. Stern-Volmer quenching plots of BSA for complexes **1**-**3**.

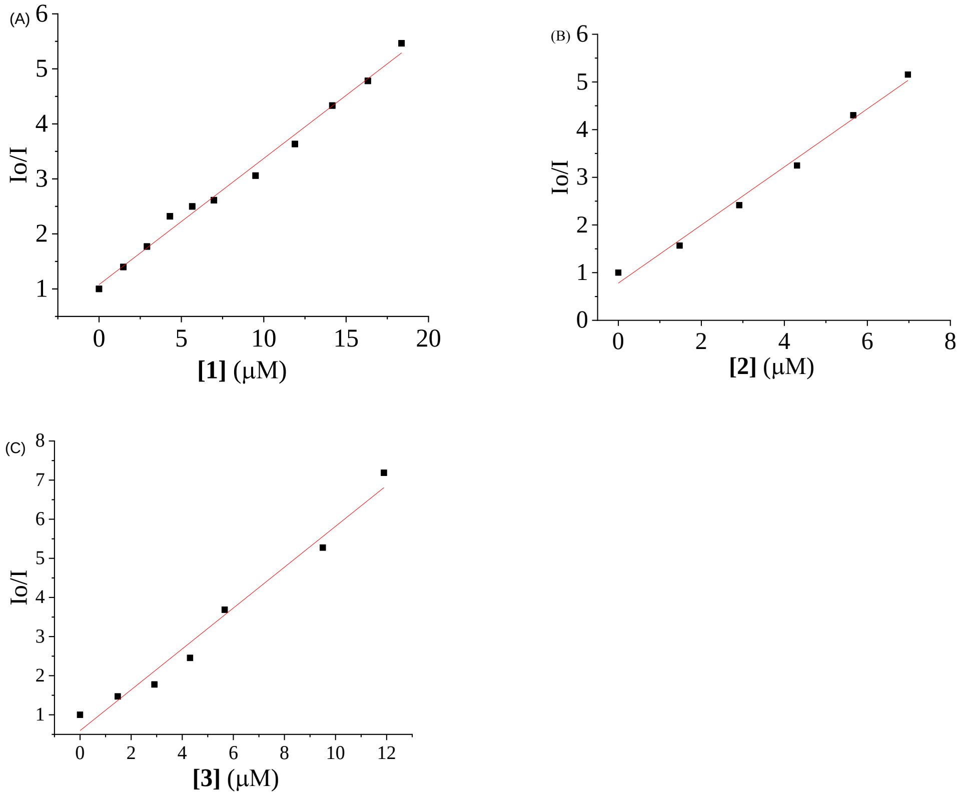

Figure S3. Stern-Volmer quenching plots of HSA for complexes **1**-**3**.

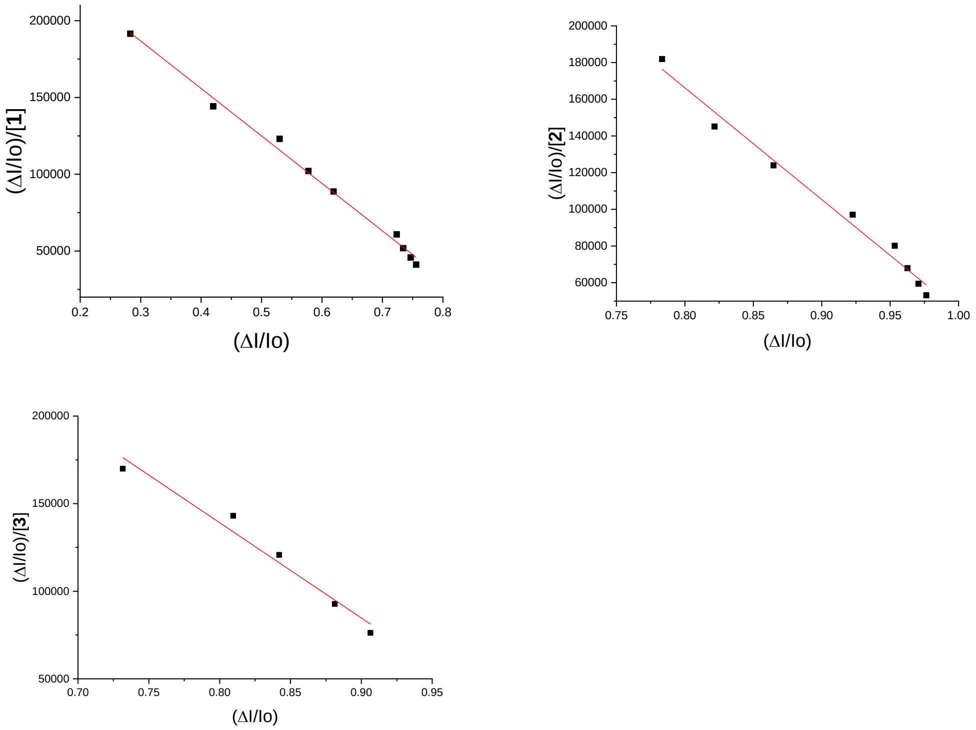

Figure S4. Scatchard plots of BSA for complexes **1-3**.

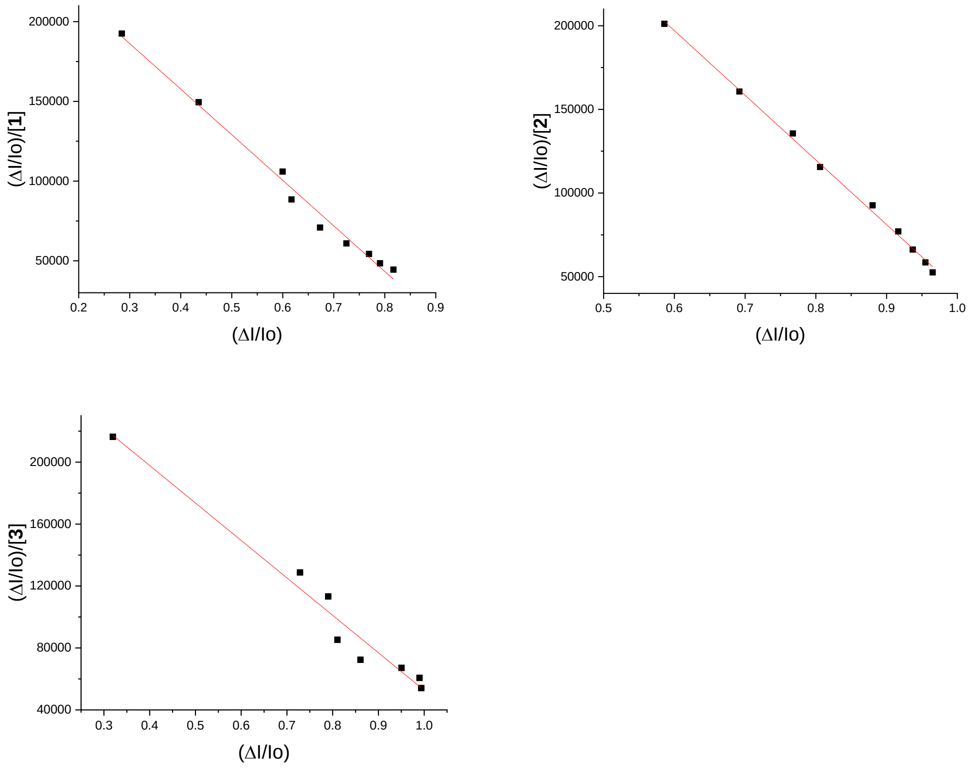

Figure S5. Scatchard plots of HSA for complexes **1-3**.

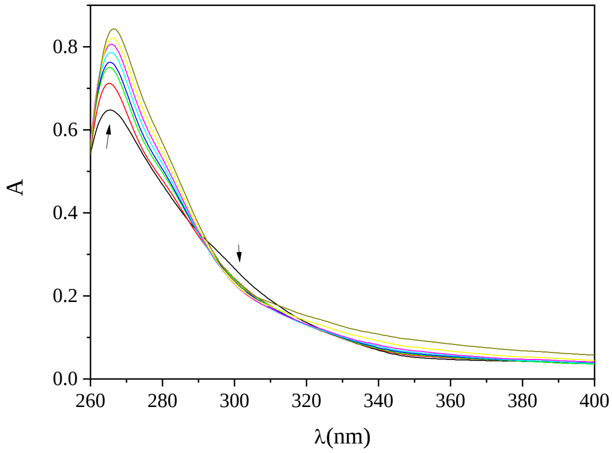

(A)

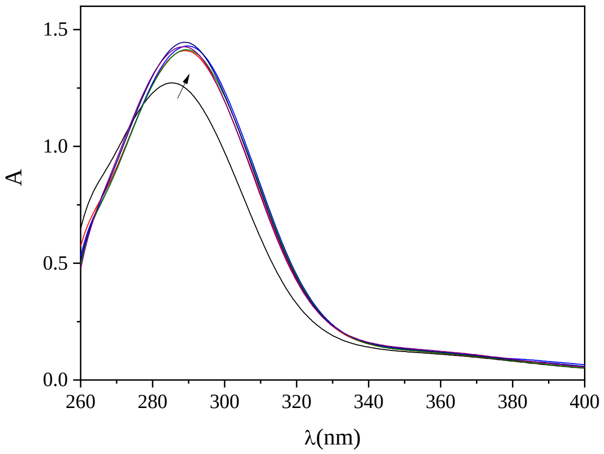

(B)

Figure S6. UV–vis spectra of a DMSO solution of (1×10⁻⁵ M) complex (A) **1** and (B) **2**, in the presence of increasing amounts of CT DNA. The arrows show the changes upon addition of increasing amounts of CT DNA.

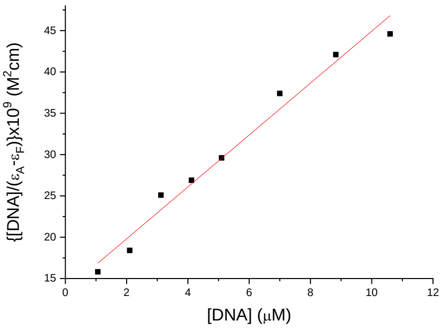

(A)

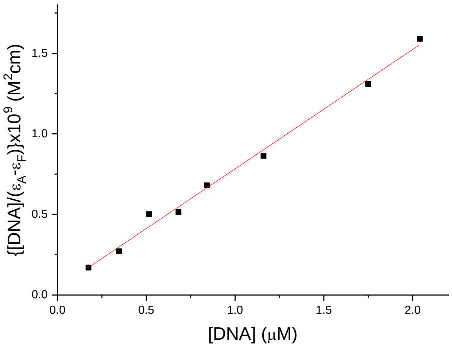

(B)

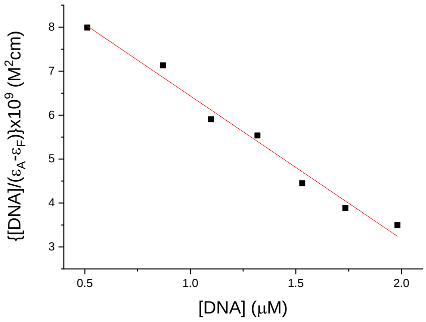

(C)

Figure S7. Plots of [DNA]/(ε_A-ε_f) *versus* [DNA] for complexes (A)-(C) **1-3**, respectively.

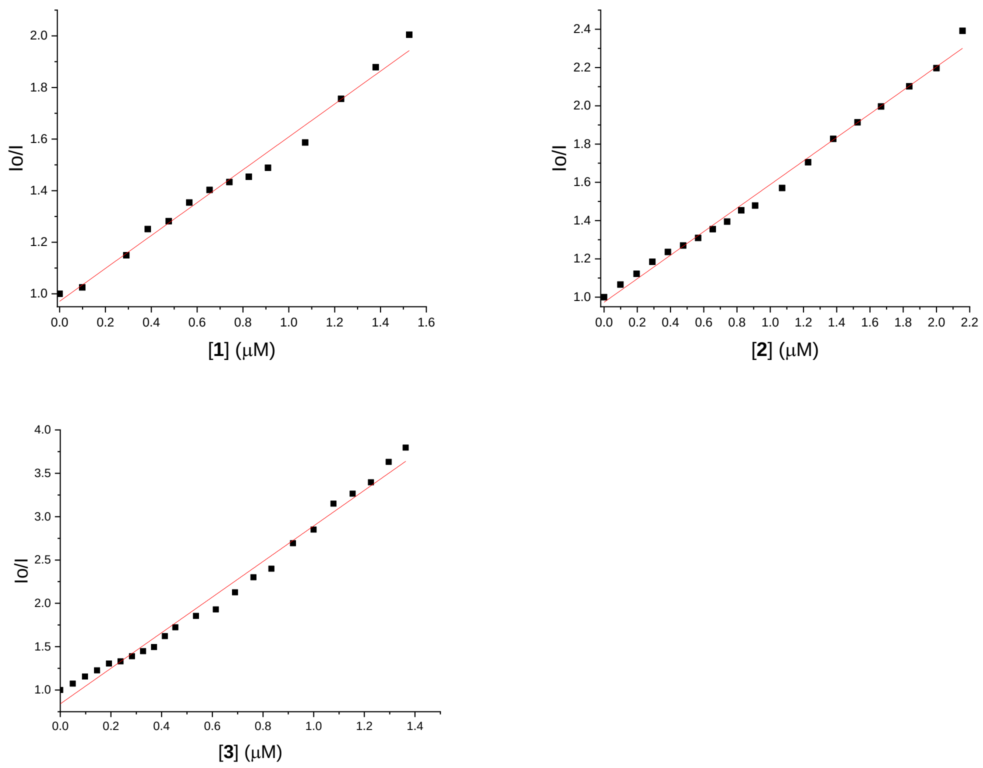

Figure S8. Stern-Volmer quenching plots of EB-DNA fluorescence for complexes **1**-**3**.
